# Lipid nanoparticles for antisense oligonucleotide gene interference into brain border-associated macrophages

**DOI:** 10.3389/fmolb.2022.887678

**Published:** 2022-11-03

**Authors:** Macarena Calero, Lara H. Moleiro, Aline Sayd, Yeray Dorca, Lluis Miquel-Rio, Verónica Paz, Javier Robledo-Montaña, Eduardo Enciso, Fernando Acción, Diego Herráez-Aguilar, Thomas Hellweg, Luis Sánchez, Analía Bortolozzi, Juan C. Leza, Borja García-Bueno, Francisco Monroy

**Affiliations:** ^1^ Department of Physical Chemistry, Faculty of Chemistry, Complutense University, Madrid, Spain; ^2^ Health Research Institute Hospital 12 de Octubre (Imas12), Madrid, Spain; ^3^ Physikalische und Biophysikalische Chemie, Universität Bielefeld, Bielefeld, Germany; ^4^ Department of Pharmacology and Toxicology, Faculty of Medicine, Complutense University, Madrid, Spain; ^5^ Centro de Investigación Biomédica en Red de Salud Mental (CIBERSAM) ISCIII. Madrid, Madrid, Spain; ^6^ Department of Organic Chemistry, Faculty of Chemistry, Complutense University, Madrid, Spain; ^7^ Institut d’Investigacions Biomèdiques de Barcelona, Spanish National Research Council (CSIC) 08036 Barcelona, Barcelona, Spain; ^8^ Institut d’Investigacions Biomèdiques August Pi i Sunyer (IDIBAPS), Barcelona, Spain; ^9^ Instituto de Investigaciones Biosanitarias, Universidad Francisco de Vitoria, Madrid, Spain

**Keywords:** perivascular/meningeal macrophages, lipidic nanoparticles, GapmeRs, mRNA, L-PGDS gene, neuroinflammation

## Abstract

A colloidal synthesis’ proof-of-concept based on the Bligh–Dyer emulsion inversion method was designed for integrating into lipid nanoparticles (LNPs) cell-permeating DNA antisense oligonucleotides (ASOs), also known as GapmeRs (GRs), for mRNA interference. The GR@LNPs were formulated to target brain border-associated macrophages (BAMs) as a central nervous system (CNS) therapy platform for silencing neuroinflammation-related genes. We specifically aim at inhibiting the expression of the gene encoding for lipocalin-type prostaglandin D synthase (L-PGDS), an anti-inflammatory enzyme expressed in BAMs, whose level of expression is altered in neuropsychopathologies such as depression and schizophrenia. The GR@LNPs are expected to demonstrate a bio-orthogonal genetic activity reacting with L-PGDS gene transcripts inside the living system without interfering with other genetic or biochemical circuitries. To facilitate selective BAM phagocytosis and avoid subsidiary absorption by other cells, they were functionalized with a mannosylated lipid as a specific MAN ligand for the mannose receptor presented by the macrophage surface. The GR@LNPs showed a high GR-packing density in a compact multilamellar configuration as structurally characterized by light scattering, zeta potential, and transmission electronic microscopy. As a preliminary biological evaluation of the mannosylated GR@LNP nanovectors into specifically targeted BAMs, we detected *in vivo* gene interference after brain delivery by intracerebroventricular injection (ICV) in Wistar rats subjected to gene therapy protocol. The results pave the way towards novel gene therapy platforms for advanced treatment of neuroinflammation-related pathologies with ASO@LNP nanovectors.

## 1 Introduction

Neuroinflammation occurs in the central nervous system (CNS) in response to exposure to diverse types of stress, either physical, psychological, or mixed (Garcia-Bueno et al., 2008). It is considered a protective mechanism aimed to restore the structural and functional integrity of the inflamed organ. However, neuroinflammation may become deleterious in severe, non-controllable, and/or long-lasting conditions, as reviewed in Sochocka et al. (2017). In line with its two-faced nature, neuroinflammation has been identified as a core element in the etiopathophysiology of several neurological and neuropsychiatric diseases (Schain and Kreisl, 2017; Yuan et al., 2019). The duration and degree of neuroinflammation should be precisely regulated by compensatory anti-inflammatory pathways. One of these mechanisms involves the synthesis of cyclopentenone prostaglandins, such as 15-deoxy-PGJ_2_ (15d-PGJ_2_), an endogenous ligand of the nuclear receptor peroxisome proliferator-activated gamma (PPARγ), which exerts anti-inflammatory, anti-oxidant, anti-excitotoxic, and pro-energetic effects in the brain (Garcia-Bueno et al., 2008). 15d-PGJ_2_ is a non-enzymatically dehydrated product of prostaglandin D_2_ (PGD2). PGD2 is formed from the common precursor of the prostanoid prostaglandin H2 (PGH_2_) by the action of the enzyme lipocalin-type prostaglandin (PG) D synthase (L-PGDS). Constitutive L-PGDS expression has been found in brain–blood interfaces such as the choroid plexus, particularly by CNS border-associated macrophages generically known as BAMs (Pedragosa et al., 2018; Kierdorf et al., 2019). As distinguishable by their specific localization at the CNS interfaces that populate a constant replacement rate, BAMs can be classified either as perivascular macrophages (PVMs) or as meningeal macrophages (MGMs) (Urade et al., 1993; Vasilache et al., 2015; Urade, 2021). The two cell types, PVM and MGM, belong to a common non-parenchymal myeloid lineage that derives from the same erythromyeloid progenitor (Goldmann et al., 2016). Their strategical position at the CNS barriers suggests similar functions as sentinels against infections and tissue damage (Herz et al., 2017). The BAMs form part of the “neurovascular unit,” in conjunction with other cellular types such as vascular endothelial cells (ECs), neurons, astrocytes, myocytes, pericytes, and extracellular matrix components (Iadecola, 2017).

The concept of the neurovascular unit is still evolving, as well as its (patho)physiological roles, couplings, and regulatory mechanisms (Schaeffer and Iadecola, 2021). Whether peripheral inflammatory signals, for example, cytokines, prostaglandins, or structural components of bacteria, such as lipopolysaccharide (LPS) or lipoteichoic acid (LTA), can reach the brain after stress exposure and, thus, cause neuroinflammation despite the walled defense by the brain–blood barrier (BBB) is still an open debate (Dantzer et al., 2008). Several complementary and non-excluding pathways have been proposed: A) the neural pathway, involving systemic cytokines directly activating primary afferent nerves such as the vagus nerve; B) the humoral pathway, affecting the choroid plexus and circumventricular organs, which physiologically lack an intact BBB. These leaky regions may be the access points for circulating pro-inflammatory cytokines to enter the cerebral parenchyma by volume diffusion and elicit downstream signaling events, which are important in altering brain function; C) the cellular pathway, which implicates systemic inflammation in association with both activation of ECs of the cerebral vasculature and an increase in circulating monocytes with a possible infiltration to brain parenchyma. Systemic pro-inflammatory cytokines activate ECs, expressing receptors for the pro-inflammatory cytokines TNF-α and IL-1β, which, in turn, signal to PVMs and MGMs strategically located adjacent to ECs.

Because of the central role of BAMs in neuroinflammation (both PVMs and MGMs) and their phagocytic activity in the CNS, pharmacological interest has been focused on them to target neurotherapeutic compounds in the brain parenchyma (Azodi and Jacobson, 2016; Glass et al., 2010). As a relevant precedent of drug delivery exploiting, the pro-apoptotic drug clodronate encapsulated in mannosylated multilamellar liposomes has been used for BAM selective depletion (Van Rooijen and Sanders, 1994). Consequently, targeting BAMs and their pathways that contribute to neuroinflammation has the potential to be used in therapeutic approaches. Nevertheless, contributions of BAMs to (patho)physiology are quite unknown along with their developmental, molecular, and functional differences with parenchymal microglia—the other cellular type of phagocytes resident in the brain (Janda et al., 2018; Kim et al., 2021). The specific localization of BAMs in the neurovascular unit suggests, indeed, functional differences with microglia that need to be further explored. Furthermore, the number and pro/anti-inflammatory profile of BAMs have been both shown to be susceptible to change in different pathological conditions. Particularly, high-anxiety mouse strains (129S2/Sv mice) presented an increased number of activated PVMs (MHCII+), both under control and LPS conditions (Li et al., 2014). Increased PVM numbers have also been found in the postmortem brain samples of schizophrenia and depressed patients who committed suicide (Schnieder et al., 2014; Torres-Platas et al., 2014; Cai et al., 2020; North et al., 2021).

BAM genetic modulation is emerging as a promising therapeutic strategy (Prinz et al., 2021). One interesting approach is gene silencing, particularly the use of antisense oligonucleotides (ASOs) as therapeutic tools for neurodegenerative and neuropsychiatric disorders (Southwell et al., 2012; Bortolozzi et al., 2021; Doxakis, 2021). ASOs are short synthetic stretches of single-stranded DNA, usually 15–20 bp in length. The antisense sequence can hybridize with the sense sequence of the target mRNA; in a way, such binding of a specific ASO sequence prevents translation of the target. Consequently, the expression of the affected protein in the disorder is reduced, leaving a potential therapeutic effect related to gene modulation that is more precise than interventions with chemical drugs, many of which are not selective. ASO-based therapy is, indeed, an active area of gene drug development designed to treat a variety of gene-specific diseases (Zhang et al., 2011; Kole et al., 2012), especially orphan diseases (Li et al., 2018; Aoki and Wood, 2021), and definitely interesting in real settings for precision medicine (Frazier, 2015; Novak et al., 2018; Quemener et al., 2020).

**FIGURE 1:**
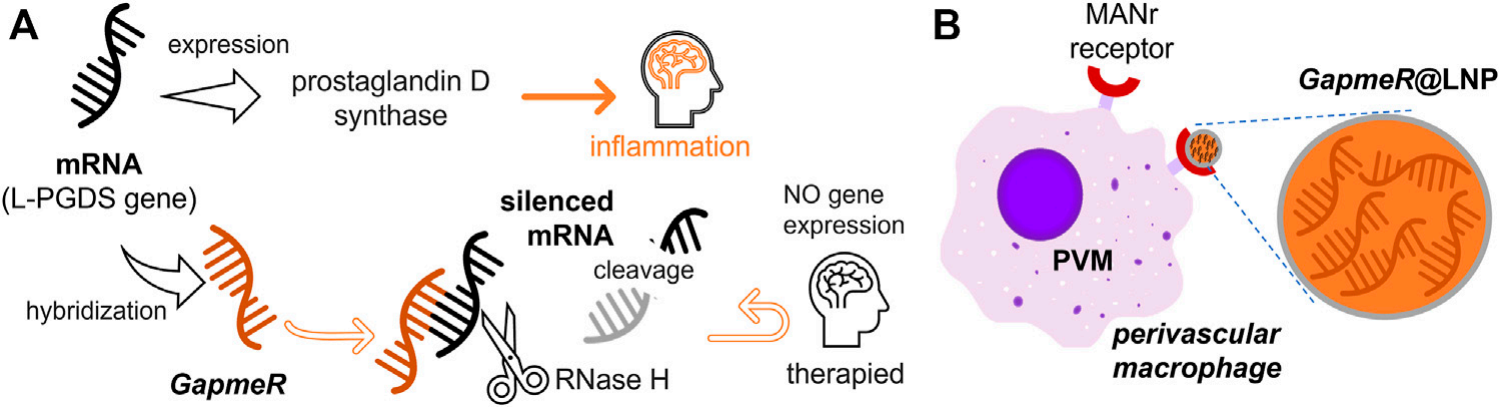
Gene therapy rationale conceived to silence mRNA expression for the L-PGDS gene in the border-associated macrophages (BAMs) responsible for prostaglandin D-synthase expression. **(A)** Therapeutic circuit. The gene-silencing route is based on the hybridization of the L-PGDS-mRNA transcript by specific antisense oligonucleotides called GapmeRs (aka GRs), which were designed for silencing *via* RNase H cleavage of the targeted gene. **(B)** GR delivery into BAMs. The GR payload is compacted into lipid nanoparticles (LNPs); these are GR@LNPs, which were designed to best incorporate a functional mannosylated formulation into GR-loading macrophages, specifically charging GR@LNPs by their surface mannose receptors.

In this work, we take advantage of bio-orthogonal nanotechnologies for the effective access of ASOs to brain-blood interfaces by means of targeted BAMs. We have provided a nanotechnological proof-of-concept based on a colloidal synthesis that incorporates ASO cargoes into lipid nanoparticles (@LNPs) at high payload compaction (Kulkarni et al., 2018). To equip an ASO with drug-like properties superior to more labile RNAs, chemical modification is required for stabilization against ribonuclease degradation (Roberts et al., 2020). We exploit second-generation ASOs, known as GapmeRs, (GR) in commercial presentations (Crooke et al., 2021). A GR exploits ribonuclease H (RNase H), a non-sequence-specific enzyme that catalyzes RNA hydrolytic cleavage if immobilized in DNA strands (Ausubel et al., 2006). For interfering with the expression of the neuroinflammatary mediators, we utilized a specific GR-family that matches the mRNA transcript for the L-PGDS gene to be silenced. Such mRNA interferential constructs are synthetized from ethyl-constrained nucleotides acting as high-affinity ribonuclease-resistant sequences (LNAs) (Braasch et al., 2003), which silence mRNA expression through inhibitory interference (Hammond et al., 2000; Elbashir et al., 2001; Hammond et al., 2001). The considered GRs contain a single-stranded oligo-DNA flanked by ribonuclease-resistant LNAs. The LNA parts increase the affinity for the mRNA target and confer nuclease resistance. The DNA moiety was designed to complementarily bind with the mRNA transcript to be interfered with for the purpose of gene silencing. In addition, this DNA part activates RNase H cleavage of the targeted RNA transcript by the endogenous enzyme (Ausubel et al., 2006). Because RNase H selectively degrades only the specific mRNA in the complementary mRNA/DNA hybrid, the considered antisense GRs will be used as specific gene silencers by the high selective cleavage of the L-PGDS mRNA transcript (see schematics in Figure 1A. In this study, we designed a GR-based gene therapy for regulating neuroinflammation in a @LNP platform implemented in BAMs (Figure 1A). We aimed at silencing the gene that encodes the L-PGDS enzyme, a multifunctional protein constitutively expressed in BAMs. As a functional pillar of our therapeutic nanovector, the GR-loaded LNPs, shortly named GR@LNPs, were manufactured as BAM-vectorizable vehicles (Chen et al., 2019; Hou et al., 2021). The new GR@LNPs could be particularly efficient in treating neuroinflammation if incorporated into BAMs involved in endogenous defense mechanisms. The specific BAM-targeting was designed to bind the CD206 mannose receptor presented by the macrophage surface (Umezawa and Eto, 1988). Some previous works have already taken advantage of mannosylated liposomal drugs charged into BAMs (Buiting et al., 1996; van Rooijen and van Kesteren-Hendrikx, 2002); the mannosylated liposomes were formulated with the macrophage clearing agent dichloromethylene bisphosphonate, named clodronate (CLO). The delivery tactic consisted of incorporating cytolytic CLO into macrophages at the targeted tissue to later release the drug under cell lysis (van Rooijen and van Kesteren-Hendrikx, 2002). In our nanotechnological approach, to the best association with gene-modulated BAMs able to affect the neurovascular unit in the BBB, the GR@LNPs are engineered for highest GR payload compared to the previous CLO-based approaches. Also BAM adhesion is optimized, as provided by the specific ligand p-aminophenyl-α-D-mannopyranoside (Kong et al., 2012), hereinafter referred to as MAN. This MAN ligand will be conjugated onto the GR@LNP surface to enhance brain delivery as compared to the suicidal CLO-tactic (Sayd et al., 2020). Taking advantage of the neurofunctional BAMs, our novel nanotechnological rationale is depicted in Figure 1B as a bio-orthogonal design to silence the L-PGDS gene at conserving cellular integrity. The mannosylated GR@LNP carriers are vectorized to BAMs as associated through their surface MAN receptors. Our bio-orthogonal synthesis route has been designed *via* a multi-step colloidal assembly with Bligh–Dyer solvents for optimal GR compaction into engineered LNPs. The synthetized GR@LNP nanovectors were subjected to preclinical evaluation of gene-silencing activity in functional BAMs of Wistar rats. We used a well-established validation *in vivo* setting based on intracerebroventricular (ICV) administration, specifically designed for targeting the mannose receptor (CD206) as presented by the BAM surface (Polfliet et al., 2001a; Galea et al., 2005; Sayd et al., 2020).

After years of preclinical research on ASO/GR overwhelming toxicological troubles and regulatory limitations (Frazier, 2015), only a few ASO/GR nanotechnological constructs have been marketed with a clinical efficacy (Huggett and Paisner, 2017). Furthermore, fabrication of enhanced lipid-vectored ASO/GR nanomedicines requests on bio-orthogonal chemistries (Han et al., 2021; Horejs, 2021; Zhu et al., 2022). In the last 2 years, lipid nanoparticles changed the history in the form of COVID-19 vaccines based on mRNA (Hou et al., 2021; Janjua et al., 2021; Witwer and Wolfram, 2021). The successful history of lipid nanoparticles fulfills today, at last, the promise of nanotechnology to revolutionize drug delivery in the forthcoming years (Scioli Montoto et al., 2020; Huang et al., 2022). A molecular settlement of the ASO/GR-based gene delivery and lipid nanoparticle topic—*where nano meets bio*—must be, hence, established to further navigate the nanotechnological realm from the engineering desk, through the synthesis bench, up to the clinics. This work explores the molecular nano–bio interface for the optimized nanomaterial design, aiming to obtain reliable lipid-based GR nanovectors as potentially applicable for clinical neurology gene therapy. Further understanding the involved colloidal interactions could help foster translation toward the clinical setting. The article is consequently organized as follows: we first reviewed the theoretical background involved in our bio-orthogonal colloidal engineering as grounding the nanotechnological rationale needed to fabricate the novel GR@LNP nanovectors for BAM delivery. The Experiment section is organized into two parts: 1) Materials and Methods, including the (bio)chemicals used to synthetize the mannosylated GR@LNPs and their physicochemical characterization methods; 2) Evaluation *in vivo*: preclinical setting, describing the biological procedures for the validation of the GR@LNPs, as directed into functional BAMs using model Wistar rats. The Results section contains the original research occurring sequentially: 1) Synthesis protocol; 2) Physicochemical characterization; and 3) Evaluation *in vivo*. The Discussion section first includes terms on biological chemistry and gene delivery nanotechnology in relation with the biological circuitry involved in BAM targeting. We further discussed the pharmaceutical/medical aspects of gene neurotherapy, on which BAM-targeted ASO nanovectorization using mannosylated @LNP delivery platforms could imply an advance. What limitations challenge their practical implementation in the clinical setting are also discussed. Emphasis is made on discussing how a rational physicochemical design based on a profound understanding of the bio–nano interface could assist in these advances and improvements. Finally, we summarize the Conclusion.

## 2 Theoretical background: Nanotechnological rationale for the colloidal design of GapmeR nanovectors

### 2.1 Physicochemical engineering: Lamellar self-assembly under catanionic lipid condensation on GR polyanions

**FIGURE 2:**
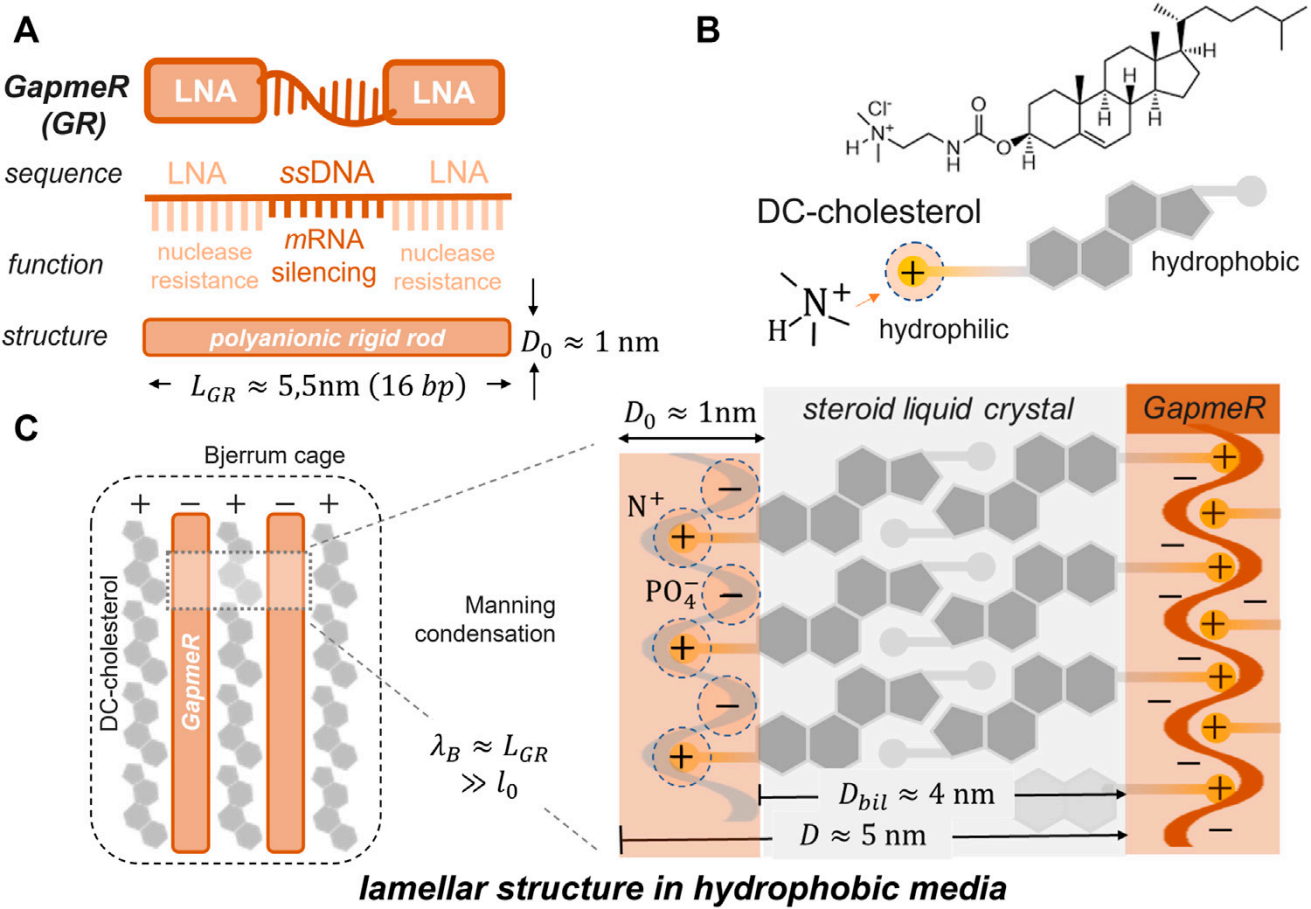
Physicochemical rationale for GapmeR (GR) compaction into lamellar liquid crystal cholesteric phases composed by DC-cholesterol (DChol) under high density Coulombic condensation. **(A)** Molecular GR construct as a 16 base-pair oligonucleotide; LNA-flanked, single-stranded DNA with the L-PGDS gene-silencing sequence (see Materials for details). Despite electrolytic dissociation of the nucleic acid phosphates ( $pK_{a}\approx 2$ ), each GR strand appears globally with the electrostatic structure as a rigid rod polyanion; coarse-grained dimensions (16 base-pairs): $L_{GR}\approx 5,5\;nm$ length ( $l_{0}\approx 0.34nm$ per bp); $D_{0}\approx 1\;nm$ diameter. Topological GR parameters (per reactive anionic group (PO_4_^−^); 1bp): specific rod area $A_{0}^{GR}\approx {\pi D}_{0}l_{0}\approx 1{nm}^{2}$ ; laterally exposed area per anion $a_{0}^{GR}\approx \pi l_{0}^{2}\approx 0.4{nm}^{2}\ll A_{0}^{GR}$ ; and nominal charge density $\sigma_{GR}=-e/A_{0}^{GR}\approx -e/{nm}^{2}$ . **(B)** Amphiphilic structure of the DC-cholesterol (DChol) molecule, as constituted by the steroidal hydrophobic moiety linked to a hydrophilic counterpart by a tail terminated in the cationic quaternary dimethylammonium head group (Me_2_N^+^–), endowed of a large cross-sectional area ( $a_{0}\approx 1{nm}^{2}$ ) and a strong basic characteristic ( $pK_{a}\approx 9.3$ ). Approximated molecular dimensions (for DChol chloride; M.W. 537.3 g mol^−1^; solid density $\rho_{0}\approx 1\;g/{cm}^{3}$ ): specific volume $v_{0}\approx 0.9{nm}^{3}$ ; Me_2_N^+^–head group area $a_{0}^{DChol}\approx 0.5{nm}^{2}$ ; nominal charge density $\sigma_{DChol}=+e/a_{0}^{DChol}\approx +2e/{nm}^{2}$ ; head-to-tail molecular length $D_{1}\approx 2nm$ ; and aspect ratio $\alpha =v_{0}/a_{0}l_{0}\approx 0.9$ , predicting a cylinder-like aspect; bilayer former). **(C)** GR-DChol lipoplexing as promoted at high-compaction inside Bjerrum cages in a low-permittivity dielectric medium (left panel); the Bjerrum length is assumed to have a size comparable to the charged rod length ( $\lambda_{B}\approx L_{GR}$ ), much larger than the elemental charge size ( $\lambda_{0}\gg l_{0}$ ). A lamellar GR-DChol structure promoted by Manning condensation under steroidal mesogenicity is proposed (right panel). *Expected topological lipoplex stoichiometry:* $a_{0}^{GR}\approx a_{0}^{DChol}$ and $\sigma_{DChol}\approx -2\sigma_{GR}$ , suggesting a chemical formula as two DChol per GR with a bilayer organization for the DChol molecule. Because of the strong acid–base reactivity between GR and DChol, a catanionic 2:1 stoichiometry is assumed under the Coulombic assembly underlying the lamellar bilayer structure (GR: DChol_2_). *Lamellar lipoplex dimensions:* The anionic component condensates into parallel GR-monolayers, with a thickness of $D_{GR}\approx D_{0}\approx 1\;nm$ , which are electrostatically “cemented” by intercalated bilayers made of cationic DChol, with a thickness of $D_{bil}\approx {2D}_{1}\approx 4\;nm$ . The resulting lamellar spacing expected in Manning-condensing media is expected; therefore, $D\approx D_{GR}+D_{bil}\approx D_{0}+{2D}_{1}\approx 5nm$ , in agreement with experimental ultrastructural observations (in Figure 4).

**FIGURE 3:**
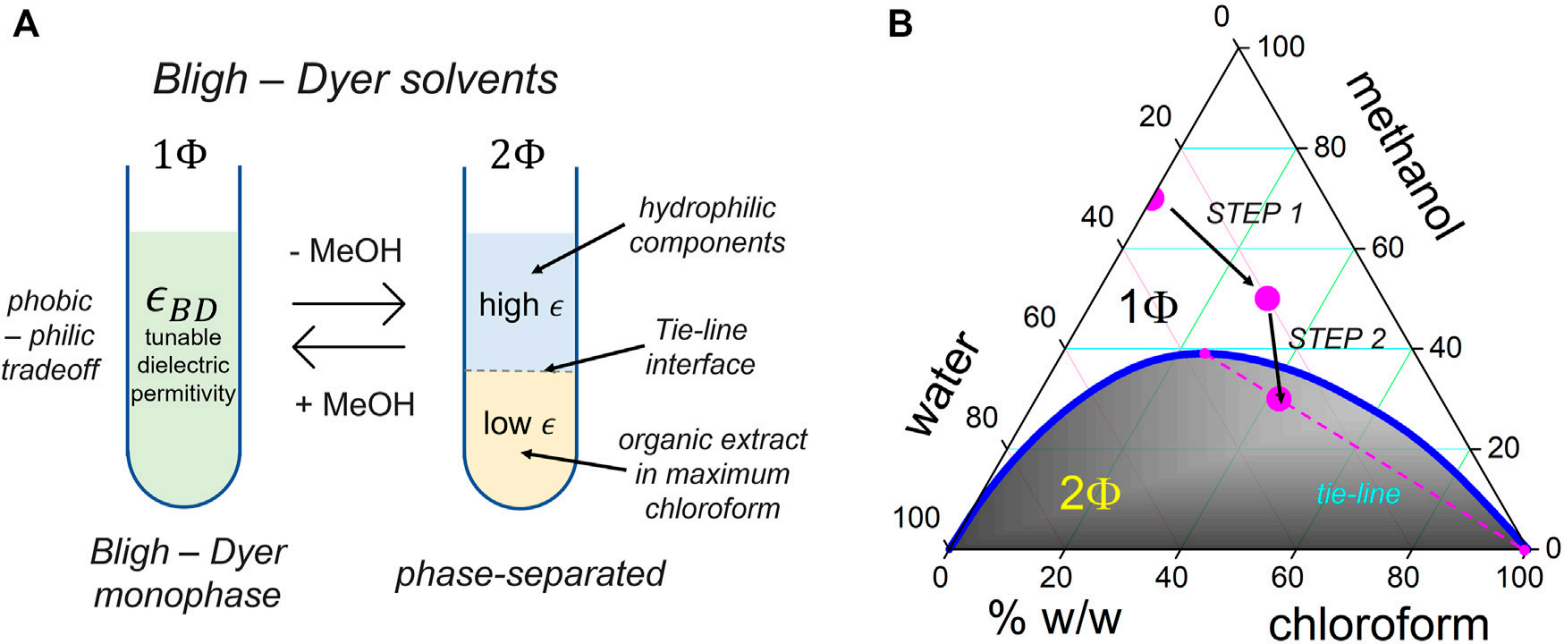
**(A)** Bligh–Dyer (BD) tunable solvents as modulators of the phobic–philic trade-off controllable under a compatibilizer (methanol: MeOH), which enables for tuning the dielectric BD permittivity ( $\epsilon_{BD}$ ). The canonical BD solvent is a monophase that combines oil (chloroform) and water with a content of MeOH compatibilizer (1Φ), close or higher than a critical composition (ca. 55% chloroform; 30% water; and 15% methanol). BD mixtures with a poor content in methanol segregate into two phases (2Φ); the rich-in-water, high-permittivity BD solvent containing hydrophilic components (light phase; upper) and the “maximum” chloroform, low-permittivity BD phase that allows for extracting the most hydrophobic components (heavy phase; lower). The material exchange of components happens through the “tie-line” interface, which stabilizes the spinodal decomposition between the BD phases upon depleting the BD compatibilizer (MeOH). **(B)** Phase BD diagram as constituted by the ternary mixture H_2_O/CHCl_3_/MeOH. The liquid percentages are expressed in weight per weight (% w/w), as adapted from the original article (Kebbekus et al., 1995). The blue line represents the phase separation (1Φ→2Φ) binodal boundary. We followed a two-step procedure to transfer GR and DChol from water-rich media into a low-permittivity electrostatic environment with a highly condensing (Manning-like) lipoplexing capacity (see Figure 2, and explanation in main text for details). Phase segregation occurs as spinodal decompositions along tie-lines. We chose directions toward a maximum chloroform line in the biphasic region (magenta dashed line), which allows partitioning of hydrophilic components into the aqueous upper layer (essentially water–methanol), and hydrophobic, non-polar, neutral components such as most lipids and GR-DChol lipoplexes into an organic phase as a chloroform-rich heavy layer. Both layers are colloidally nanostructured close to the critical point (Kebbekus et al., 1995), a fact here exploited to the best extraction efficiency of the most condensed components into drop-templated nanoparticles. The organic “maximum chloroform” BD layer is an efficient host for extracting the neutral catanionic aggregates, independent of their intrinsic hydrophilicity, whereas the water-rich layer requires less cost in free energy to insert non-complexed GRs and non-aggregated polar components.

The ASO chains, in general, and the GR sequences considered in this study, take a rigid conformation as linear polyanion strands with the phosphate groups exposed to the outer helix surface (Uppuladinne et al., 2019). As a construction principle, we invoke the concept of counterion Manning-like condensation around the polynucleotide chains, behaving as rigid rods at a high anionic charge (Kebbekus et al., 1995). Figure 2 shows the supramolecular catanionic assembly composed of cationic DChol as condensed into the GR polyanion (Kim et al., 2008). The structure of the GR-polyanion is described in Figure 2A, and the DChol component as the condensation counterpart is described in Figure 2B. A lamellar condensed (GR/DChol bilayer) structure resulted from electrostatic interactions, as modulated in solvents with a variable dielectric permittivity (see Figure 2C). The Bligh–Dyer (BD) solvent was used to provide a tunable permittivity medium, as composed by ternary mixtures of water, methanol, and chloroform (see the phase diagram in Figure 3B). Electrostatics and hydrophobicity are both counterbalanced by tuning BD permittivity, hence resulting in catanionic GR:DChol_2_ lipoplexes stoichiometrically formed upon a thermodynamic philic–phobic trade-off (Bligh and Dyer, 1959).

### 2.2 Components and reactivity: Catanionic lipoplexes

The GR components were chosen by their biological functionality for gene silencing and their chemical affinity as strong acids ( $pK_{a}\approx 2$ ) for the electrostatic nano-assembly. Figure 2A shows the GRs containing a central block of single-stranded deoxyribonucleotides for silencing L-PGDS-mRNA under RNase H cleavage. This ssDNA is flanked by 2′-O-methyl-modified ribonucleotide wings (LNA™), which shield the internal block from nuclease degradation (Monia et al., 1993). Coarse-graining as linear polyanions results in rigid GR rods with approximate cylindrical dimensions (for n=16 bases; width D≈1nm , length $L=nl_{0}\approx 5.5nm$ , and $l_{0}\approx 0.34\;nm$ the average size of a single base) (Alberts et al., 2015). Due to the large persistence of the ribonucleotide chains ca. 72nm≫L (Kebbekus et al., 1995), they stretch out as charged cylinders with a uniform surface density of charge $\sigma_{GR}\approx -ne/\pi LD\approx -e/{nm}^{2}$ (being −e the charge of the electron (Uppuladinne et al., 2019)); see caption of Figure 2A for details. The basic DC-cholesterol (DChol) was chosen for cationic lipid formulation under Coulombic attraction and chemical affinity for anionic nucleic acids. Furthermore, DChol entails colloidal amphiphilicity and biocompatibility superior to other cationic lipids used in drug delivery (Chen et al., 2019). Figure 2B draws its chemical formula showing the hydrophilic modification terminated by a quaternary ammonium cation head group with a cross-sectional area sized ca. 0.5 nm^2^. This is bound to the hydrophobic steroid moiety through a flexible hydrophilic linker. The head-to-tail length of this amphiphile is estimated to be ca. 2 nm, which assigns a cylindrical aspect ratio ( 1/2≪α≈0.9 ), thus being susceptible for the lamellar assembly through liquid crystal interactions as planar bilayers (Oswald and Pieranski, 2005; Israelachvili, 2011). The chemical affinity of DChol by the single nucleotides stands up on their high basicity in aqueous solutions ( $pK_{a}\approx 9.3$ ), accomplishing the acid–base complexation reaction

$$GR+2\;DChol\;⇌\;G\mathrm{R:}{DChol}_{2}$$

at 1:2 lipoplex stoichiometry underlying structural bilayer relationships, i.e., $a_{GR}=a_{DChol}$ and $n_{GR}={2n}_{DChol}$ (assuming each single nucleotide reacting as an equivalent GR-unit with the two molecules of DChol that constitute the bilayer). The electrostatic neutrality condition follows as $n_{GR}a_{GR}+n_{DChol}a_{DChol}=0$ ; see caption in Figure 2 for details.

### 2.3 Control of dielectric permittivity: Bligh and Dyer solvents

We exploited the Bligh–Dyer (BD) concept for hydrophobic extraction in modulated solvents, the gold standard for natural lipid extraction (Kebbekus et al., 1995). As described in Figure 3, our synthesis approach was implemented into a multistep BD-schema at modulated control of dielectric permittivity ( ϵ ). Figure 3A depicts the adaptation of the BD concept for optimized catanionic complexation, including the reaction, further compaction, and final extraction in homogeneous phases as composed by BD solvents. Briefly, catanionic GR–DChol interactions have become highly condensing under low dielectric permittivity in such a way that mixing double-excess DChol with GR yields catanionic GR:DChol_2_ lipoplexes. The resulting hydrophobic homogenate is transferred into a biphasic BD system with a modulated phobic/philic trade-off under permittivity control; however, the lowest permittivity BD phase concentrates the GR:DChol_2_ lipoplexes with the highest hydrophobicity, and the non-aggregated GR polyanions can be recovered in the high-permittivity phase. As composed by ternary mixtures of water (H_2_O), methanol (MeOH), and chloroform (CHCl_3_), the BD solvents were modulated under thermodynamic control; see the phase diagram in Figure 3B. The proposed BD procedures are fast, efficient, reproducible, and easy to implement (Kebbekus et al., 1995). By using greener solvents, the proposed catanionic complexation in BD solvents is potentially scalable up to pharmacologically enhanced standards translatable to human medicine (green organics, fluorinated oils, eutectic solvents, *etc.* (Breil et al., 2017)). As a featured piece of physicochemical understanding on the proposed synthetic procedures for catanionic lipoplexing, we further discuss the control mechanisms for solvation, reaction, and condensing interactions generically involved in BD solvents as follows.

### 2.4 Electrostatic control of lipoplex interactions: Bjerrum’s cage

The spatial strength of the catanionic lipoplex assembly was evaluated with respect to disentangling thermal fluctuations. In particular, the Bjerrum length ( $\lambda_{B}$ ) determines the distance above which Coulomb’s interactions weaken with respect to thermal energy ( $k_{B}T$ ). In BD media with a tunable dielectric permittivity ( $\epsilon_{BD}$ ), the Bjerrum length is given as follows:

$$\lambda_{B}\equiv \frac{e^{2}}{\epsilon_{BD}k_{B}T},$$

which is defined relative to the vacuum value ( $\epsilon_{0}$ ; in electrostatic units $4\pi \epsilon_{0}\equiv 1$ ), where $k_{B}$ is the Boltzmann constant and T is the absolute temperature.

The Bjerrum length determines the size of a systemic “binding cage” in which catanionic bonds dominate over thermal forces (see Figure 2C; left panel). Coulombic interactions are expected to be short-ranged in hydrophilic BD solvents ( $\epsilon_{BD}\approx \epsilon_{w}\approx 80\epsilon_{0}$ ; thus, $\lambda_{w}\approx 0.01\lambda_{0}$ ). However, the Bjerrum cage is much smaller, thus weakly condensing in water ( $\lambda_{w}\approx 0.7\;nm$ ) than extremely condensing in vacuum ( $\lambda_{0}\approx 60\;nm$ ), or in organic-rich BD solvents ( $\epsilon_{org}\approx 5\epsilon_{0}\approx 0.05\epsilon_{w}$ ; thus, $\lambda_{org}\approx 20\lambda_{w}$ ). Hence, the dielectric BD compatibilizer (methanol) appears as the key order modulator regulating the Bjerrum’s cage for strengthening the catanionic assembly in organic-rich phases (because $\epsilon_{MeOH}\approx 30\epsilon_{0}$ , intermediate between water $\epsilon_{w}\approx 80\epsilon_{0}$ , and chloroform $\epsilon_{org}\approx 5\epsilon_{0}$ , then $\lambda_{w}\ll \lambda_{MeOH}<\lambda_{org}\approx \lambda_{0}$ ). Consequently, catanionic condensation becomes optimal in the lowest permittivity BD solvent (maximum chloroform), which imparts the highest electrostatic ordering inside the largest Bjerrum cage ( $\lambda_{org}\approx 15nm\gg \lambda_{w}$ ). On the contrary, electrostatic condensation weakens in water-rich BD solvents ( $\lambda_{w}\approx 0.7nm$ for $\epsilon_{w}\approx 80\epsilon_{0}$ ).

### 2.5 Tunable Manning condensation of GR/DChol lipoplexes in BD solvents

The GR polyanions are initially diluted in water, where the Bjerrum length is significantly smaller than the distance between the neighboring nucleotides ( $\lambda_{w}>l_{0}\approx 0.34nm$ ); here, they repel each other, thus making a conformationally persistent strand (the Kuhn length $l_{p}\approx 70nm\gg \lambda_{w}>l_{0}$ ) (Kebbekus et al., 1995). Because Coulomb interactions strengthen the considered GR polyelectrolytes $\Gamma \equiv \lambda_{w}/l_{0}\approx 2.1$ (Manning, 1969), we thus predicted a GR stretching out, followed by favored counterion condensation as dissolved in aqueous media; for a single GR polyanion in the presence of cationic lipid counterparts in water, we expect a condensation fraction $\theta \approx 1-\Gamma^{-1}\approx 0.5$ (Manning, 1969). In the absence of cationic lipid (DChol), however, Manning’s condensation is extremely low ( θ≈0 ), becoming only partial at a low electrolyte concentration below a critical value ( $\sigma <\sigma_{c}$ at Γ>1 ) (Hayes et al., 2015). For the GR:DChol_2_ lipoplex catanions considered at high dilution, we expect θ≥0.53 ( Γ≈2.1 ), implying that at least 53% of phosphate groups are condensed within the Bjerrum length. At moderate environmental hydrophobicity (e.g., in the BD monophase $\epsilon_{BD}\leq \epsilon_{w}$ ), a single counterion layer of DChol cations is expected to condense inside the Bjerrum cage, with a modulated size compatible with each single GR chain ( $l_{0}\ll \lambda_{BD}\approx 16l_{0}\approx 5nm$ ), akin an inverse micellar aggregate. In more concentrated GR systems, however, higher electrostatic screening is needed with increasing ionic strength, which requests higher environmental hydrophobicity for Manning condensation to happen (Dobrynin and Rubinstein, 2005). Mean-field theoretical approaches predict higher condensation than estimated beyond the Manning threshold (Schiessel and Pincus, 1998; Schiessel, 1999), provided the freely mobile small counterions are considered within the Debye–Hückel approximation (Na^+^, K^+^, *etc.*); these theories predict a vanishing density of charge ( $\sigma_{c}\to 0$ ) (Stigter, 1995), in total condensation conditions ( θ→1 ) (Hansen et al., 2001). Even at a high polyelectrolyte density but at low enough permittivity media, attractive counterion-mediated interactions are known to induce complete condensation (Ha and Liu, 1998; Podgornik and Parsegian, 1998).

### 2.6 Catanionic lipoplex reaction in lamellar structures

The molecular ordering Manning interactions are expected to drive liquid crystal-like DNA condensation (Koltover et al., 2000; Muñoz-Ubeda et al., 2010). In concentrated GR/DChol phases, catanionic condensation could eventually happen in the “maximum chloroform” BD solvent (at $\epsilon_{org}\ll \epsilon_{w}$ , thus $\lambda_{org}\approx 15nm\gg \lambda_{org}$ ). The rigid GR polyanions could, thus, condense into catanionic GR:DChol_2_ lipoplexes, with a planar rigid conformation and large size in a BD medium of low enough dielectric permittivity (see Figure 3). In addition, DChol is highly mesogenic (Oswald and Pieranski, 2005), with a high propensity for liquid crystalline catanionic condensation within GR into multilamellar arrangements with a hydrophobic steroidal core and exposed cationic surfaces at a high density of ammonium GR-binding groups (see Figure 2C for a molecular depiction; later contrasted with experiments). Because mesogenicity favors charge polarization on the outer DChol-surfaces, straightforward surface-guided Manning condensation should be able to reverse the interactions between polyanion chains from being repulsive to attractive (Ha and Liu, 1998; Podgornik and Parsegian, 1998). Those mesogenic Manning-like condensations induce a transition into a lamellar phase with the neutralizing 1:2 stoichiometry (under the chemical formula GR:DChol_2_). These expectations correspond to the regime of high density and high (physiological) ionic strength, thereby holding charge neutralization in sandwiched catanionic layers; here, the resultant neutralized charge density holds $\sigma_{GR}a_{GR}+\sigma_{DChol}a_{DChol}=\sigma a\to 0$ (having $a\approx 0.5{nm}^{2}$ the GR-to-DChol condensation area). In the pursue for optimal catanionic condensation into neutral lipoplexes (GR:DChol_2_), which occurred under the lamellar self-assembly into lipid nanoparticles (GR@LNPs), we expect matching electrostatic neutralization leading to a vanishing zeta potential ( ζ→0 ).

### 2.7 GapmeR containing lipid nanoparticles (GR@LNPs): Chemical formulation

The lipid nanoparticles (@LNPs), as carriers of nucleic acids, particularly ASOs (GRs), are the most advanced delivery systems used so far in genetic medicines (Thi et al., 2015; McKay et al., 2020). In this work, we designed the synthesis procedure of the GR@LNPs to fulfill three principal terms: 1) to protect the GR payload at high compaction; 2) assist with cell uptake through the plasma membrane; and 3) release the GR payload into the cytosol at the highest transfection efficiency and the lowest toxicity as possible. Based on the aforementioned specifications, our formulation should include the following: a) ASO(GR), as an active pharmacological agent; b) cationic DChol lipid for ASO(GR) compaction under Manning condensation at 2:1 stoichiometry; c) unsaturated phospholipid POPC, a bilayer former as a lipid stabilizer; d) lipid helper cholesterol, as a hydrophobic filler and stability enhancer; e) PEGylated phospholipid DSPE-PEG, as a surface cell adhesion additive (Noiri et al., 2019); f) functional phospholipid DSPE-PEG-MAN for specific anchoring to the mannose receptor of BAMs (MANr), selectively from associated microglia that do not express MANr (Galea et al., 2005). The wanted lamellar phase represents the densest state achievable inside the solid-like @LNP-core with a stoichiometry, corresponding to two lipid counterions (DChol) per base pair (i.e., GR:DChol_2_). The GR@LNPs were formulated with a functional shell for specific and selective adhesion to BAMs, as depicted in Figure 1B. The chemical formulation that embodies the engineered GR@LNPs is summarized in Table 1.

**TABLE 1:** Chemical formulation for lipid nanoparticles based on the condensed lamellar GR/DChol phases, as engineered in this work. Each component is considered by their physiological function and structural contribution to the formulation (see main text). The condensed GR/DChol phase, together with undetermined amounts of interstitial cholesterol, is assumed to constitute the LNP core. The molar ratios of each shell component are referred to the DChol molar concentration chosen as a reference. The other lipid components were chosen for mainly partitioning the LNP shell as composed lipid bilayers. The lipid formula refers to the neutral lipids considered to form the shell membrane; these are considered apart from DChol, the core forming cationic lipid.

Component	Acronym	M.W. (g mol-1)	Lipid formula (%mol)	DChol ratio	Physiological function	Physicochemical interaction	Site
GapmeR (ssDNA/LNA)	GR	16 bases	N/A	**0.5**	Nucleic acid pharmacological payload	Polyanion charge	Core
**DC-cholesterol**	**DChol**	**537.3**	N/A	**1**	**Inert**	**Cationic lipid counterion**	Core
Cholesterol	Chol	386.7	30%	**0.3**	Inert	Helper lipid/hydrophobic structural enhancement	Core/shell
Palmitoyl-oleyl-phosphocholine	POPC	760.1	60%	**0.4**	Cell membrane interaction	Membrane stabilizer/fluid bilayer former	Shell/core
PEGylated phospholipid	DSPE-PEG	ca. 2810	1%	**0.01**	Cell adhesion lipid	Outer membrane stability and adhesion enhancer	Shell
Mannosylated PEGylated lipid	DSPE-PEG-MAN	ca. 3000	9%	**0.05**	Binding to mannose surface receptors in PVMs	Outer membrane PVM-specific adhesivity	Shell

*Bioactive payloads: L-PGDS gene-silencing GRs.* The active payload of the GR@LNPs was made up of catanionic GR:DChol_2_ lipoplexes; two types of single-stranded *antisense* oligo-GRs were chosen with a L-PGDS silencing activity: **GR1)** 14–16pbs (5′-3′-TAC​TCT​TGA​ATG​CAC​T) and **GR2)** 14–16pbs (5′-3′-AGT​TAC​ATA​ATT​GCC​A). As a negative control (no gene interference), we used a random *no-sense* sequence: **GRc)** 14–16pbs (5′-3′-AAC​ACG​TCT​ATA​CGC). These GRs are labeled at the 5′ end with a fluorescein-derived isomer being emitted at 488 nm (6-FAM and 6-carboxyfluorescein).

*@LNP formers: structural lipids.* Cationic DNA-complexing DChol was chosen as a biocompatible lipid as it can pack the nucleic acid payload into lipoplexes (Muñoz-Ubeda et al., 2010). By mimicking membrane-forming components (Alberts et al., 2015), the rest lipid formula for the vehicle assembly is as follows: a) structural POPC (60%); b) rigidizing cholesterol (30%); c) surface adhesion lipid DSPE-PEG (1%), and d) functional lipid DSPE-PEG-MAN (9%) for specific adhesion to MAN-receptor of BAMs (indicated as molar percentages). Our colloidal synthesis plan takes advantage of this chemical formulation within the nano-assembly concepts discussed earlier; these are as follows: a) the catanionic (acid–base) stoichiometric reaction modulated by dielectric permittivity in BD solvents and b) tunable Manning-driven aggregation for controlled lipid extraction and compatibilization.

## 3 Experiment: Fabrication of GR@LNPs targeted to BAMs

### 3.1 Materials and methods

#### 3.1.1 Chemicals

1-Palmitoyl-2-oleoyl-sn-glycero-3-phosphocholine (POPC) and 3ß-[N-(N′,N′-dimethylaminoethane)-carbamoyl]cholesterol (DChol) were purchased from Avanti. 1,2-Distearoyl-sn-glycero-3-phosphoethanolamine-N-polyethyleneglycol (DSPE-PEG; 2 kD PEG M.W.) and its succinimide-functionalized counterpart 1,2-distearoyl-sn-glycero-3-phosphoethanolamine-N -[succinimidyl (polyethylene glycol)] (DSPE-PEG-NHS) were obtained from NOF EUROPE GmbH (Japan). Cholesterol (Chol), p-aminophenyl-α-D mannopyranoside (MAN), and methanol (MeOH) were supplied by Sigma-Aldrich (Germany). LNA™ *antisense* DNA-oligos (GRs) and *no-sense* oligo-DNA were obtained from Exiqon® (United States). Clodronate (CLO) was from the Clodrosome® macrophage depletion kit commercialized by Encapsula NanoSciences®. Chloroform (CHCl_3_) and RNAse-/DNAse-/protease-free water were purchased from Acros Organics (Belgium/United States). Phosphate buffer saline (PBS) was obtained from Gibco (United Kingdom). All the other solvents and reactants were from Merck-Sigma. Solvent densities ( $\rho_{i}$ in g/cm^3^) are as follows: 1.0 for water ( i=1 ); 0.8 for methanol (MeOH; i=2 ), and 1.5 for chloroform (CHCl_3_; i=3 ). The Bligh–Dyer (BD) solvent is based on ternary mixtures prepared by volumetry (volume fractions $\phi_{i}=v_{i}/\sum_{i}v_{i}$ ; weight fractions $w_{i}=\phi_{i}\rho_{i}/\sum_{i}\phi_{i}\rho_{i}$ for i=1, 2, 3 ). All chemicals, solutions, glassware, and plasticware were sterilized in an autoclave prior use, stored at 5°C, and strictly handled in a biosafety cabinet in sterile conditions.

#### 3.1.2 Chemical synthesis of the MAN-lipid surface ligand: Mannosylation reaction

As a specific PVM ligand, we synthetized the mannosylated lipid DSPE-PEG-MAN using a click chemistry reaction method accomplished by following a previously reported procedure described for the mannose precursor p-aminophenyl-α-D-mannopyranoside, shortly named MAN (Tan et al., 2012). This synthesis starts with a covalent bonding between the succinimide-functionalized PEG-lipid (DSPE-2000PEG-NHS; 50 mg, 1 equiv.) and mannose precursor (5.4 mg, 1.2 equiv.) as the reaction occurred in THF (6 ml). The reaction mixture was stirred at room temperature, and the reaction advanced, followed by thin layer chromatography. After 6 h reaction time, the solvent was removed under vacuum, the raw product was dissolved in CHCl_3_, and filtered to remove excess of the unreacted MAN precursor. The reaction product was used without further purification and characterized by following the standard spectroscopic techniques. The final yellowish oily product was stored at 5°C and considered usable for 3 months. To find out whether the mannosylation product remained stable during this time, a 1H-NMR spectrum was performed for the mannosylated lipid synthetized DSPE-PEG-MAN (or MAN-lipid). The proton NMR spectrum reported in Supplementary Figure S2 shows three mannose-specific proton signals, appearing preserved after covalent binding of the MAN precursor. They specifically correspond to sugar MAN protons at large chemical shifts around a characteristic phospholipid resonance at 7.25 ppm (Alexandri et al., 2017).

#### 3.1.3 L-PGDS gene-silencing GapmeR (GR)

We used commercial GRs, specifically designed to target the L-PGDS gene at highly efficient inhibition (Exiqon, United States; see Chemicals). They contain a central stretch (gap) of single-stranded DNA flanked by blocks of LNA™-modified nucleotides (by patent to Exiqon^®^). The LNA™ blocks increase the target affinity and nuclease resistance of the oligo, whereas the DNA gap activates RNase H cleavage of the target mRNA (L-PGDS gene transcript) upon binding. The considered GRs are 14–16 RNA nucleotides in length and fully phosphorothioated. An enhanced pharmacokinetics of LNA™-based GRs has been demonstrated in different therapeutic approaches (Petersen and WengelLNA, 2003). LNA™ antisense oligonucleotides are well-tolerated and show low toxicity *in vivo*. In addition, short, high-affinity LNA™-GRs are active at lower concentrations than other ASOs (Petersen and WengelLNA, 2003; Davis et al., 2006; Campbell and Wengel, 2011).

#### 3.1.4 Dynamic light scattering

Particle sizes and size distributions (polydispersity) were evaluated by laser dynamic light scattering (DLS), using a 90Plus/BI-MAS particle size analyzer (Brookhaven Instruments Ltd.). Measurements were performed at 25°C, in samples constituted by diluting about 0.1 ml of LNP suspension in 2.9 ml of PBS buffer, at least in triplicate. The mean values were accumulated after 5 min readout time. By exploiting the Stokes–Einstein relationship using standard approaches, the apparent particle sizes were calculated from the particle diffusion coefficient as the hydrodynamic radius obtained from the diffusional times of the DLS autocorrelation functions for the given value of the viscosity of the solvent; this is $R_{H}$ (Brown et al., 1975; Chu, 1976). The measured polydispersity index corresponds to the standard deviation of the experimental size distribution defined as $PDI=1+\sigma_{R}/R_{H}$ .

#### 3.1.5 Zeta potential

An indirect measurement of the surface LNP charge was carried out in dilute LNP suspensions by measuring the zeta potential ( ζ ), using a NanoBrook ZetaPALS analyzer that exploits phase analysis light scattering under electrophoretic particle migration (Brookhaven Ltd.). The NanoBrook instrument is suitable for measurements at very low electrophoretic mobilities in dilute suspensions using special cuvettes with the addition of 1 mM of KNO_3_. Such measurements cover the range of typically ±100 mV corresponding to mobilities as low as 10^–8^ m^2^/V s. The NanoBrook ZetaPALS analyzer covers this range and amplifies by a factor of ×1000 in sensitivity. The electrophoretic mobilities were evaluated, at least in triplicate, after 5 min readout time. The calculated mean values were then related to the zeta potential using the Smoluchowski relationship (Hunter 1981). The net surface potential of the LNP is estimated as ζ , which can be associated with a surface density of charge ( σ↤ζ ).

#### 3.1 6 Transmission electron microscopy

The nanoscale structure of the LNPs was analyzed by transmission electron microscopy (TEM), performed with a JEOL instrument (100 keV JEOL-1010 FXII microscope), working at 0.35 nm spatial resolution. For TEM examination, the samples were dispersed in PBS buffer (0.1 mg/ml) and then incubated in uranyl acetate (UO_2_^2+^ at 2% w. w.) for 1 min for staining. One drop of the diluted suspensions was poured on Formvar carbon-coated copper grids, allowing the solvent to evaporate at room temperature. The imaging camera used was MegaView II (Soft Imaging System, United States). Image analysis was performed by ImageJ software.

#### 3.1.7 Pharmacotechnical design

A new pharmacological GR presentation was designed to be carried out at lipid nanoparticles, named as GR@LNPs. We adapted mild methods of colloid chemistry for the bio-orthogonal synthesis of GR@LNPs at the top GR payload, high cargo protection and stability, global isotonicity, and chemical neurocompatibility. The synthesis is performed at room temperature in PBS buffer (described as follows as a main result in a protocol). The obtained GR@LNPs match *in vitro* with the osmotic pressure in the blood so that it does not increase the intracerebral volume *in vivo*. The novel GR@LNP nanovector prototypes were engineered to bring the European market under the Spanish Patent ES2698565B2; they were designed to steer the chain of the value from pharmaceutical manufacturing, preclinical testing, translational and logistic complexities, and strict regulations, up to eventual adaptation to clinical trials.

## 4 Evaluation *in vivo:* Preclinical setting

Adult male Wistar Hannover rats (HsdRccHan:Wist, from Harlan, Spain), weighing 350–400 g (N = 15), were housed in cages from the start of the protocol (n = 3–4) and maintained at a constant temperature of 24 ± 2 °C at a relative humidity of 70 ± 5% in a 12 h light–dark cycle (lights on at 8:00 a.m.). The animals were fed with standard pellet chow (A04 SAFE, Scientific Animal Food and Engineering©, Augy, France) with free access to fresh tap water and were maintained under constant conditions for 7 days prior to experiments. All experimental protocols were approved and followed the guidelines of the Animal Welfare Committee of the Universidad Complutense of Madrid (PROEX 419/15), according to European legislation (2010/63/UE). Animal studies are reported in compliance with the ARRIVE guidelines, and all efforts were made to minimize animal suffering and to reduce the number of animals used.

### 4.1 Intracerebroventricular (ICV) administration

For assessing the targeting activity of the GR@LNP nanovectors to deliver GRs in the BAMs adjacent to the intracerebroventricular (ICV) spaces, an ICV injection was chosen as a validated administration setting. The ICV injection of mannosylated liposomes is a well-established procedure to specifically target the mannose receptor (CD206)-expressing BAMs without affecting resident microglial numbers in both rats (Polfliet et al., 2001a; Newman et al., 2005; Sayd et al., 2020) and mice (Galea et al., 2005; Hawkes and McLaurin, 2009). These studies demonstrated that effective targeting of BAMs required the mannosylated liposomes to be administered directly into the cerebrospinal fluid (CSF). Given the unidirectional CSF flow, the maximal exposure of the brain surface to GR@LNPs in the CSF is necessarily accomplished by the ICV injection, as described in Galea et al. (2005). The GR@LNP uptake efficiency was evaluated into BAMs with respect to the reference control constituted by clodronate-containing liposomes (CLO#GR@Ls), as based on the commercial CLO preparation, previously validated to reach BAMs using the ICV injection (Clodrosome^®^ macrophage depletion kit dissolved in PBS at 5 mg/ml) (Sayd et al., 2020). For this purpose, six rats were anesthetized with an intraperitoneal injection of ketamine and xylazine (2.5:1 mg/kg, SC). They were then mounted on the stereotaxic ICV frame. We used a 26-GA needle guided through the right lateral ventricle (1 mm posterior to Bregma, 0.25 mm lateral to the midline, and 3.5 mm ventral to the surface of the skull), with a motorized stereotaxic ICV injector (Stoelting^®^ 53,311). The GR-based preparations (either commercial CLO 5 mg/ml or PBS buffer at pH 7.4) were respectively infused to three animals in a volume of 25 μL over 10 min using a Hamilton syringe (Bonaduz, AG Switzerland^®^). CLO-induced BAM clearance was evaluated under CD163+ depletion, as detected by immunofluorescence. As expected, there was a lack of immunosignals for the cellular marker CD163^+^ (ED2) in parenchymal vasculature and meninges in CLO animals compared to PBS-treated rats; see previous results in Sayd et al. (2020).

### 4.2 *In vivo* assessment of L-PGDS silencing by GR@LNPs

Adult male Wistar Hannover rats were divided into three groups (N = 9; three per group): a) the control group, receiving LNPs without GR (only the vehicle); b) the negative control group, receiving GR@LNPs with no-sense GR3 (non-complementary to the RNA fragment coding for L-PGDS); and c) the positive control group, receiving GR@LNPs with *antisense* GR1 and GR2 (complementary to RNA fragments coding for L-PGDS). The animals were anesthetized and placed on a stereotaxic frame, and different GR@LNPs were administered by ICV. The GR@LPN preparations were slowly diffused into the right lateral ventricle of the rat brain over 10 min *via* a motorized stereotaxic injector at a dose of 25 μL GR@LNP preparation (the total GR concentration estimated at 0.15 nmol/μL). One week after injection, the animals were euthanized with an overdose of pentobarbital, and immediately after the brain was removed, it was frozen on dry ice and stored at –80°C for subsequent histological preparation of tissue samples. To identify the BAM route and evaluate the silencing activity of the GR@LNPs, a histological characterization was performed as follows.

### 4.3 Immunofluorescence studies: Confocal imaging

The rat brain was cut by using a cryotome into 15-μm thick sections. In order to detect the BAM marker CD163, the sections on coverslips were washed three times for 5 min with 0.02 M KPBS and incubated in a blocking solution (10% bovine serum albumin and 0.1% Triton X-100 in 0.02 M KPBS) for 60 min at room temperature. Once removed from the blocking solution, the sections were incubated with antisera for a mouse monoclonal anti-CD163 (ED-2) antibody (sc58965, 1:200; Santa Cruz Biotechnology^®^) during 2 h at room temperature. Subsequently, the sections were washed with KPBS five times during 5 min each and then incubated for 2 h at room temperature with Alexa Fluor^®^ 555-conjugated donkey antimouse IgG (h + l) highly cross-adsorbed secondary antibody (A-31570, 1:1000; Life Technologies^®^). The sections for confocal studies were washed in KPBS five times for 5 min each and then blocked for 30 min with 10% BSA and 0.1% Triton X-100 in KPBS. Then, the sections were incubated 1 h at room temperature with antisera for a mouse antirat RECA-1 antibody (MCA970R, clone HIS52, 1:1000; BioRad^®^). Subsequently, the sections were incubated for 0.5 h at room temperature with IRDye^®^ 680R goat antimouse 926-68070 (D10512-15, 1:1000, Li-Cor). The sections were washed in 0.02 M KPBS five times for 5 min. For microglial immunofluorescence, antigen retrieval was performed over a second set of sections with a solution of sodium citrate at pH 6.0 during 40 min, ranging from 40°C to 65°C. The sections were washed three times for 5 min with 0.02 M KPBS and incubated in the blocking solution (10% bovine serum albumin and 0.1% Triton X-100 in 0.02 M KPBS) for 60 min at room temperature. Then, they were incubated with antisera for a rabbit polyclonal anti-ionized calcium-binding adapter molecule 1 (Iba1) (ab108539, 1:100; Abcam^®^) for 48 h at 4°C. Subsequently, the sections were washed with KPBS five times for 5 min, and then incubated for 1.5 h at room temperature with Alexa Fluor^®^ 555-conjugated donkey antirabbit IgG (H + L) highly cross-adsorbed secondary antibody (A-31572, 1:1000; Life Technologies^®^). The sections were washed in 0.02 M KPBS five times for 5 min. Finally, 4′,6-diamidino-2-phenylindole dihydrochloride (DAPI) containing Fluoroshield (Sigma-Aldrich) mounting medium was added to the slides. The sections were cover slipped and frozen at −20°C or immediately visualized using a high-performance fluorescence microscope. The confocal images were obtained by using the confocal Olympus microscope FV1200, from CAI-UCM “Centro de Citometría y Microscopía de Fluorescencia.” The images were processed for adjusting brightness, contrast, and merging images in the processing package “Fiji.” We have analyzed the brain prefrontal cortex of the three animals of the group, and two images for three consecutive brain sections of the animals have been used. The images shown are a qualitative representation of the 6-FAM immunosignal in the different groups studied. To verify whether there was non-specific binding of fluorescent secondary antibodies, negative controls were performed for each of the antibodies using the same incubation protocol without the addition of the primary antibody.

### 4.4 Mannose receptor BAM localization

Finally, a third set of sections were incubated with a 1:100 solution of the primary monoclonal rabbit antireceptor mannose antibody (CD206) (ab64693, Abcam, United Kingdom), which was maintained at 37 °C overnight. Subsequently, the sections were washed with KPBS five times during 5 min each and then incubated with a 1:300 solution of the secondary antibody goat antirabbit IgG bound to Alexa Fluor^®^ 555 (A31572, Thermo Fisher Scientific, United States) for 1 h at room temperature. Over these sections, some images were obtained using a TI-FL Epi-fl Illuminator^®^ Nikon Eclipse Ti Series epifluorescence microscope equipped with ultraviolet, blue, and green exciting filter sets and a Nikon DS-Qi1Mc monochrome camera. The following filters were used to visualize the fluorescence signal of probes: UV for DAPI (exc/em: 358/461 nm), blue (exc/em: 495/517 nm) for 6-FAM, and green (exc/em: 555/580 nm) for Alexa Fluor^®^ 555. The photographs were processed with ImageJ®x software.

### 4.5 Gene-silencing activity: *In situ* hybridization (ISH)

Coronal brain sections containing the prefrontal cortex (15 μm-thick) were obtained and processed, as described in Ferres-Coy et al. (2016). The oligodeoxyribonucleotide probe sequence, complementary to bases rat PGDS mRNA, was CTC ACC TGT GTT TAC TCT TGA ATG CAC TTA TCC GGT TGG GGC AGG (GenBank accession NM 013015.2 obtained from IBIAN Technology, Zaragoza, Spain). The oligonucleotide probe was individually labeled (2 pmol) at the 3′ end with [^33^P]-dATP (>2500 Ci/mmol; DuPont-NEN) using terminal deoxynucleotidyl transferase (TdT, Calbiochem). For hybridization, the radioactively labeled probe was diluted in a solution containing 50% formamide, 4x standard saline citrate, 1x Denhardt’s solution, 10% dextran sulfate, 1% sarkosyl, 20 mM phosphate buffer, pH 7.0, 250 μg/ml yeast tRNA, and 500 μg/ml salmon sperm DNA. The final concentration of radioactive probes in the hybridization buffer was in the same range (∼1.5 nM). The tissue sections were covered with the hybridization solution containing the labeled probes, overlaid with Parafilm coverslips, and incubated overnight at 42 °C in humid boxes. The sections were washed four times (45 min each) in a buffer containing 0.6 M NaCl and 10 mM Tris-HCl (pH 7.5) at 60 °C. Hybridized sections were exposed to a Biomax MR film (Kodak, Sigma-Aldrich, Madrid, Spain) for 24–72 h at –80°C with intensifying screens. For specificity control, adjacent sections were incubated with an excess (50x) of unlabeled probes. The films were analyzed, and relative optical densities were evaluated in three adjacent sections of the prefrontal cortex including four measurements in each section (two measurements in medial and lateral edges, respectively) as duplicate for each rat and averaged to obtain individual values using a computer-assisted image analyzer (MCID, Mering). The MCID system was also used to acquire black and white images. Image management was performed using Adobe Photoshop software (Adobe software). Contrast and brightness of images were the only variables that were digitally adjusted under expert guidance.

### 4.6 Statistical analysis

All values are expressed as the mean ± SEM. Statistical comparisons were performed by GraphPad Prism 9.0 (GraphPad software, Inc., San Diego, CA, United States) using the appropriate statistical tests, as indicated in the figure legend. The outlier values were identified by the Grubbs’ test (i.e., extreme studentized deviate, ESD method) by GraphPad Prism software and excluded from the analysis when applicable. Differences among means were analyzed by two‐way analysis of variance (ANOVA), followed by Tukey’s multiple comparison test. Differences were considered significant at *p* < 0.05.

## 5 Results

1) Colloidal Synthesis of GR@LNPs: we build upon the aforementioned engineered design for compaction of polyanion GR-rod sections under Manning (I and II) condensations by cationic DChol (together with all the other lipids). A colloidal construction was envisaged for GR@LNPs made of lamellar GR/DChol phases composed by catanionic lipoplexes (at neutralized acid–base stoichiometry GR:DChol_2_). They were templated into the emulsion droplets that were spontaneously formed with a nanometric size, net zero charge, and stable structure, as further resuspended in a physiological buffer. These nanoparticle scaffolds were designed to spontaneously assemble under BD emulsification (Bligh and Dyer, 1959), which was adapted as a phase inversion process, allowing for stable transference of compacted lamellar phases (Kheirolomoom et al., 2015).

**FIGURE 4:**
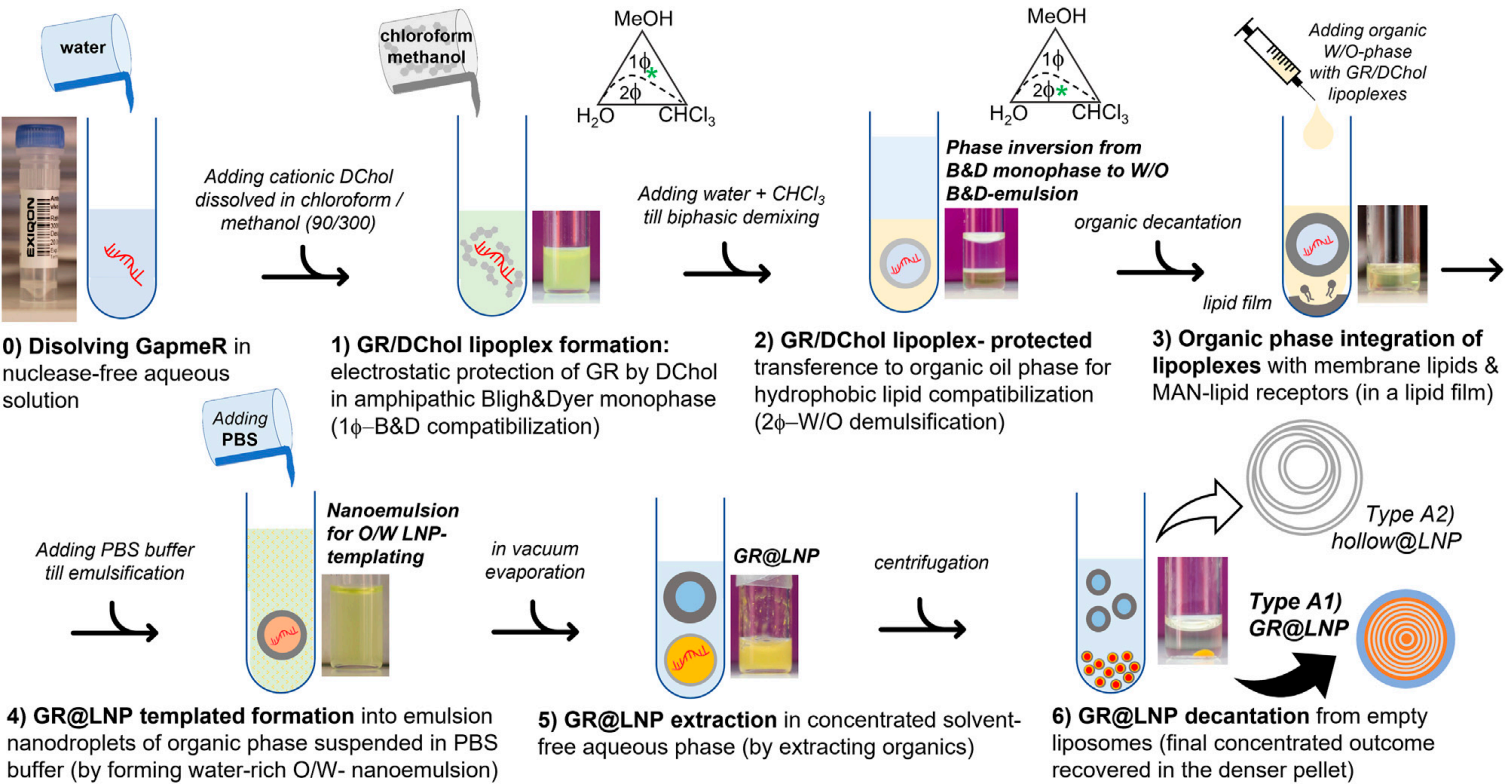
Synthesis process for the preparation protocol of GR payloads into catanionic GR-lipid nanoparticles in Bligh–Dyer media (GR@LNPs). Two fractioned outcomes are recovered from the final decantation. Step 6: *lower fraction*): denser pelleted fraction containing the synthetized GR@LNPs (*Type A1*, as characterized by TEM). *Upper fraction*): lighter supernatant containing lipid aggregates without GR being charged (*Type A2* hollow @LNPs, as characterized by TEM). Ordinals indicate the step number, as described in the main text.

Synthesis protocol for catanionic lipid nanoparticles: Figure 4 shows a schematic representation of the novel colloidal synthesis designed as a following six-step protocol:

Phase A) GapmeR protection by complexation with DC-cholesterol.

Step 0) GapmeR (GR) solution and DC-cholestrol solution (DChol): A) GR solution was prepared by adding 90 μL of endonuclease-free water to the plastic tube containing 40 nmol GRx (x = 1 and 2 or c); the total amount of the nucleotide monomer obtained was 1.3 μmol. The tube was closed, and the contents were gently agitated until complete dissolution of GRs took place. B) A solution of DChol in chloroform was prepared (90 μL CHCl_3_) in a glass vial at a concentration of 18 mg/ml; then, the solution was mixed with 300 μL methanol. Final concentrations resulted into 7 mM of total GR nucleotides (0.45 mM GR) and 8 mM of DChol. Critical step: The work was carried out in a biological cabin to avoid for exposition to environmental nucleases. These solutions were used immediately.

Step 1) GR lipoplex formation: The aqueous GR solution was added (90 μL A) dropwise to the glass vial with DChol solution (390 μL B), both prepared in Step 0. As considered the expected stoichiometric proportion (GR:DChol2), this corresponded to slightly excess DChol (3.0 μmol), with respect to the added GR monomers (1.3 μmol). Critical step: The solution was agitated continuously upon adding to avoid local enrichments. The solution was immediately vortexed at 600 rpm for 1 min and left for incubation overnight. The GR/DChol lipoplexes suspended in the homogeneous BD monophase were presumably constituted as inverse micelles (Kheirolomoom et al., 2015). The homogeneous content of this glass vial (480 μL total volume) corresponded to the 1Φ-region of the BD phase diagram at 20% water, 60% MeOH, and 20% CHCl3 in v/v percentages (ca. 20% water, 50% MeOH, and 30% CHCl3 in w/w coordinates); see Figure 4B adapted from Bligh and Dyer (1959). Pause point: The GR/DChol lipoplexes were stable in the BD monophase and were stored at –80°C for 1–2 months.

Step 2) GR lipoplex compaction: First, 120 µL of chloroform was added, and then, 120 µL of DNAse-free water was added to the monophasic emulsion, resulting from Step 1 (720 µL total volume). The biphasic BD mixture was stirred and left to separate in two phases. The resulting liquid percentages were 30% H2O, 40% MeOH, and 30% CHCl3 (volume-by-volume), equivalent to 28% H2O, 30% MeOH, and 42% CHCl3 (weight-by-weight), which correspond to a biphasic BD emulsion that segregate into two phases under spinodal decomposition as following the “maximum chloroform” tie-line (located inside the 2Φ-region of the BD phase diagram; see Figure 3B). By following coordinates in the BD phase diagram, the denser organic phase containing the GR/DChol lipoplex is chloroform-rich, practically devoid of water, whereas the aqueous phase is water-rich, thus being lighter. Critical step: ascertainment was maintained by the adding solvent in order, CHCl_3_ first to preserve high environmental hydrophobicity (assuring compacity within the lamellar catanionic phase) and H_2_O second to cause phase segregation (along the maximum chloroform tie-line). During this phase demixing step, the most hydrophobic GR/DChol aggregates were transferred to the denser, more concentrated, organic phase, whereas the lighter aqueous phase segregates as a supernatant, eventually containing non-aggregated GRs. The presence of fluorescent-labeled GR in the organic denser phase (chloroform-rich) and the total absence of GR fluorescence in the aqueous lighter phase (water-rich) were assured by visual inspection. This BD biphase was processed immediately without storage. To further concentrate the neutral GR/DChol micelles in the organic phase, the resulting demixed biphase was centrifuged for 4 min at 800 rpm. After decantation, the denser (organic) phase containing the usable nanoparticle synthesis fraction was collected using a Hamilton syringe, and the lighter (aqueous) supernatant was discarded to contain only hollow aggregates. The volume of the organic phase extracted was annotated (at least 200 µL).

Phase B) Lipid nanoparticle pre-conditioning by integration of the lipid stabilizer, lipid helper, and functional lipids with the GR/DChol lipoplex.

Step 3: Integration of the lipoplexes within lipids; lipid film formation: lipid solutions were prepared in CHCl_3_ at a final concentration of 10 mg/ml. Stabilizer lipids: POPC; helper lipid: cholesterol; and functional lipids: DSPE-PEG and DSPE-PEG-MAN (PVM-selective). Using these stocks, a lipid mixture was formed by mixing 52 µL POPC (520 µg ≡ 684 nmol), 13 µL cholesterol (130 µg ≡ 336 nmol), 4 µL DSPE-PEG (40 µg ≡ 14 nmol), and 31 µL DSPE-PEG-MAN (310 µg ≡ 104 nmol) in a glass vial, resulting at 52: 13: 4: 31 weight by weight percentages, respectively, for POPC, cholesterol, DSPE-PEG, and DSPE-PEG-MAN so that corresponding to 60: 30: 1: 9 in molar composition. The solution was gently agitated. Chloroform was eliminated under low vacuum, using a rotary evaporator (at 210 mbar and room temperature). A dry lipid film was formed at the vial bottom. The practical absence of CHCl_3_ by HPLC was verified. Lipid–lipoplex integration: the organic BD phase extracted from Step 2 with a Hamilton syringe was taken, and then, it was added dropwise in the glass vial containing the lipid film. It was incubated for 2 h to ascertain proper resuspension of the lipid components within the lipoplexes present in the organic BD phase; the solution was agitated frequently. Critical step: The complete dissolution of the lipid film was assured by gentle agitation by hand and visual inspection of solution homogeneity.

Phase C) Lipid nanoparticle templating, extraction, and purification.

Step 4: GR@LNP template formation: An equal value of PBS buffer was added as the volume of organic suspension was extracted from Step 3. It was stirred in a vortex for 1 min. A nanoemulsion was formed with the oil-in-water (O/W) structure, which acted as a scaffold for the assembly of the added lipids with the compacted lipoplexes inside organic droplets nearly 100–200 nm diameter, suspended in the aqueous phase; the droplet-templated GR@LNPs were formed by sonicating for 2 min (Kheirolomoom et al., 2015). The estimated volume of this suspension was ca. 200 μL.

Step 5: GR@LNP extraction: The organic solvents were removed from the suspension obtained in Step 4 by using a rotary evaporator at 210 mbar for 15 min. Solvent evaporation was repeated twice by rehydrating with PBS buffer (adding buffer to reach 250 µL final volume). The final GR@LNP concentration was estimated by differential weighting (ca. 3 mg/ml). The practical absence of chloroform and methanol was verified in this GR@LNP suspension by liquid chromatography (HPLC).

Step 6: GR@LNP purification: The aqueous suspension of GR@LNP was centrifuged at 14,000 rpm for 15 min. A compact pellet with the denser GR@LNPs was obtained, which was immediately decanted, weighed (nearly 1 mg dry weight), and further stored under resuspension in PBS buffer (250 µL), with the addition of vitrifying glycerol (1% w. w.). Possible lighter components such as particle-unbound lipids, hollow LNPs, and non-condensed GRs remain suspended in the remaining supernatant. The pellet containing the GR@LNP concentrate can be stored without degradation in a refrigerator at 5°C for 1–2 months. Final point: The GR@LNP concentrate can be stored at –80°C for 1 year. Once synthesis and purification procedures were outperformed, batches with GR@LNP outcomes were titrated under gravimetric estimation, characterized in physicochemical terms, and further tested for pharmacological activity *in vivo* (see Methods).

Dose gravimetric estimation: The possible losses of unbound GRs within the outcome supernatants were discarded if obtained colorless after each GR@LNP synthesis step. The initial GR payload was assumed to be completely transferred into the GR@LNP concentrate (40 nmol GR contained in 250 µL resuspending PBS buffer), provided the final GR@LNP concentrate was weighted about 1 mg after performing the aforementioned synthesis protocol, and we estimated the dosing concentration for the dispended GR@LNP preparation at approximately 0.15 nmol per µL.

### 5.1 Physicochemical characterization

Prior *in vivo* evaluation of the L-PGDS gene-silencing activity, the structure and contents of the outcome @LNP nanovectors were characterized by reference to a CLO-based @liposome formulation. The synthesis procedure was completed with three independent batches. Characterizations were performed at least in triplicate for samples on the obtained batches (experimental errors correspond to standard deviations; *t*-Student 95% confidence level; p<0.05 ). Our characterization analytics was performed as follows.

#### 5.2 Transmission electron microscopy (TEM): Microscopic textures

**FIGURE 5:**
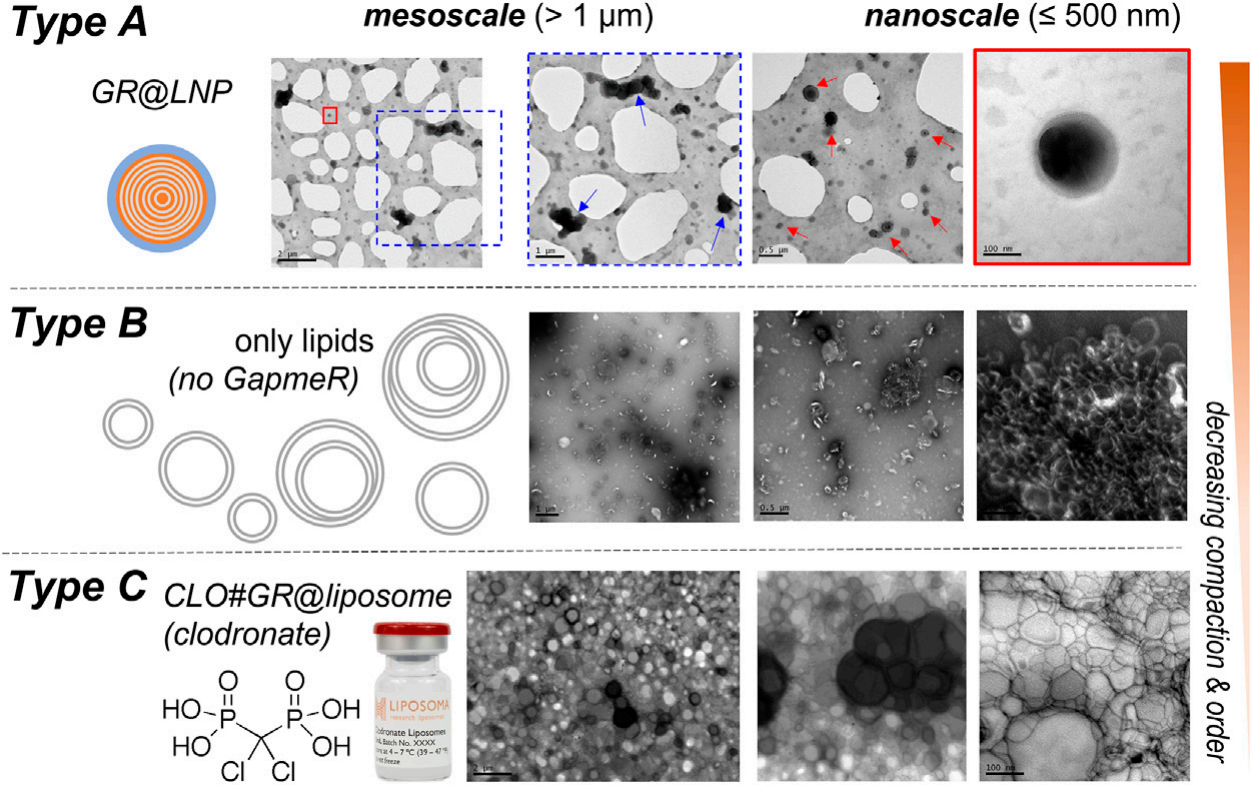
TEM characterization of the nanovector constructs considered in this work (see left panels): *Type* **(A)** GR@LNPs obtained in pelleted synthesis fraction; *Type* **(B)** hollow @LNPs obtained in the supernatant; and *Type* **(C)** CLO#GR@Ls (commercial preparate). The TEM images first show the mesoscopic textures as observed at low magnification (left-handed images; scale bars: 2 μm). TEM-images at high nanoscale magnification show the nano-objects involved in each case (right-handed images; *scale bars:* 100 nm). *Type* **(A)** colloidal synthesis *GR@LNP* outcome: The GapmeR (GR) condensed with DC-cholesterol (DChol) appeared assembled into lipid nanoparticles GR@LNPs (with the specialized lipid formulation of Table 1; synthesis protocol, as described earlier). The mesoscale textures revealed a broad distribution of sizes ranging from large aggregates (marked by blue arrows), down to the typically spherical LNPs (red arrows); these small spherical nanoparticles represent the main population of compact GR@LNP nanovectors obtained from the colloidal synthesis protocol (right panel), whereas the much larger, morphologically non-uniform aggregates constitute a minimal subpopulation disappearing after purification (a representative populational catalog is reported in Supplementary Figure S2). *Type* **(B)** hollow liposomes as obtained from the supernatant fractions obtained after synthesis. The main population corresponds to oligolamellar, mostly unilamellar, liposome specimens with a propensity for aggregation (right image). *Type* **(C)** *CLO#GR@Liposome* formulation, as obtained by mixing the used GRs with the liposomal clodronate-containing preparation used currently, as the commercial pharmaceutical standard for suicidal CNS vectorization (CLO#GR@Ls). The pristine liposomal preparation is found highly heterogeneous with a nanoscopical texture, as corresponding to a foamy material constituted by hollow, low electron density cells (swollen) surrounded by lipid membranes of higher electron density (GR-containing).

The samples of the synthetized GR@LNPs were characterized by electron microscopy. TEM grids were prepared by diluting synthesis outcomes in PBS buffer (50:50) and left for further incubation with uranyl acetate for staining (see Methods). The GR@LNPs’ samples obtained from the new synthesis protocol were comparatively studied with respect to the reaction outcome obtained in the absence of GapmeR. The commercial liposomal clodronate preparation was also characterized. Figure 5 summarizes the results of this TEM characterization. In essence, we observed a main population of near-spherical GR@LNPs with typical sizes around 100–200 nm and variable compaction at coexistence with larger heterogeneous aggregates of micrometric size, most probably formed under coalescence (Figure 5; top panels). As expected, single GR@LNPs were found to be extremely electron-dense (a high nucleotide content as revealed by uranyl contrast), with a core–shell concentric distribution of components, which will be further analyzed as follows (see Figure 5). No electron-dense compact structures but hollow liposomal aggregates were found within the synthesis outcomes devoid of GapmeR (Figure 5; central panels). This confirms the physicochemical requirement for catanionic complexation as a condition *sine qua non* for LNP compaction in hydrophobic BD media. We also prepared TEM grids for mixtures of GapmeR, with the commercial CLO-containing liposomal preparation (CLO#GR@L; see Methods). For CLO#GR@Ls, the TEM images revealed a heterogeneous liposome texture with a typical foamy aspect in the mesoscale. The main and most representative nanostructure corresponds to swollen “cells” with a hollow lumen (highly diluted interior) and adherent boundary (constituted by lipids and recruited GRs). GR compaction into electrodense cores was not detected in those CLO#GR@Ls liposomal formulations (Figure 5; bottom panels).

#### 5.2.1 Adhesion stability characterization: Mannosylation reaction

**FIGURE 6:**
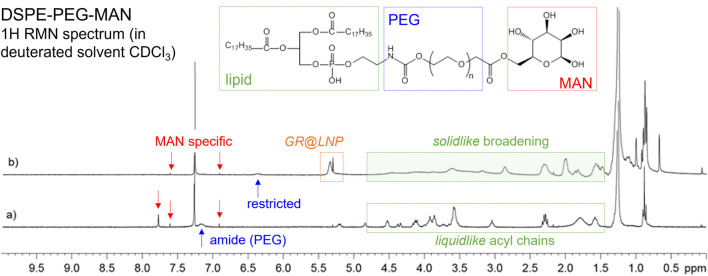
1H NMR spectra for DSPE-PEG-MAN in CDCl_3_ (300 MHz, 298 (K) for **(A)** pristine compound (chemical formula shown); **(B)** reaction mixture after GR@LNP formation. *Structural assignation (by color coding):* phospholipid moiety including acyl chains and ethanolamine heads (green); PEGylated spacer (blue); mannose ligands expected exposed in the LNPs (red). *Resonance assignation (by chemical shift):* MAN-specific resonances, as assigned in Figure 2 at 6.9, 7.6, and 7.8 ppm (red arrows); polar group PEG-amide proton exposed to the solvent in the pristine compound (at 7.15 ppm), becoming restricted (at 6.4 ppm) as the solvent protected inside the LNPs (blue arrows). A complex resonance, GR@LNP-specific resonance appears at 5.4–5.2 ppm. The characteristic liquid-like, lipid-specific 1H-resonances appeared in the 4.7–1.5 ppm interval of chemical shifts as sharp peaks, and they become strongly broaden in the LNP solid-like status (green windows).

To find out whether the mannosylated lipid DSPE-PEG-MAN occurred finally within the GR@LNPs as a BAM ligand, a proton-NMR spectrum was performed after colloidal synthesis and compared with the bare spectrum obtained for the pristine lipid obtained by chemical synthesis (see Methods). Figure 6 shows the specific mannose proton signals appeared after covalent binding of mannose occurred to the PEGylated lipid DSPE-PEG-MAN (see also Supplementary Figure S1). These genuine MAN-specific signals appeared as peaked resonances around the significant characteristic of the phospho-ethanolamine peak at 7.25 ppm (see Supplementary Figure S1).

The same MAN-specific signals were also detected without broadening in samples of GR@LNPs as obtained under the colloidal synthesis procedure described earlier (for the chemical formulation in Table 1). Remarkably, a significant mobile resonance band is observed close to the characteristic MAN structure as presumable due to a highly solvated group exchanging with the solvent (assigned to the amide proton at the diffusible linkage between the lipid moiety and the flexible PEG-chain; see Figure 6 caption). In the pristine molecule, these polar head protons exchange freely with the solvent, becoming highly restricted when the lipid moieties associate with the other LNP’s components. Indeed, GR@LNP-specific resonance is visible with a complex structure at 5.4–5.2 ppm chemical shift interval, as corresponding to highly confined protons (solid-like NMR signals). Furthermore, the complex sequence of phospholipid protons corresponding to the acyl lipid chains in the broad window of chemical shifts from 4.7 down to 1.5 ppm (Alexandri et al., 2017) was differentially observed either as sharp resonances with a typical liquid-like structure in the pristine molecule (liquidlike; Figure 6A) or strongly broaden when detected in the presence of the LNP’s components (solid-like; Figure 6B). Such structural evidence on DSPE-PEG-MAN organization, as revealed by NMR, strongly supports a functional mannosylated lipid properly organized with the highly mobile MAN group exposed to the solvent and the DSPE moiety at the restriction interaction with the much less fluid LNP’s components. As a concluding remark on this section for analytics using NMR for detecting the functional lipid DSPE-PEG-MAN, the equivalence between the MAN fingerprints found in both spectra is clearly indicative for its required presence in the GR@LNPs as the designed ligand for the mannose receptor is toward a specific nanovectorization into the brain macrophages.

#### 5.2.2 Size and polydispersity

The hydrodynamic size of the considered nanovectors was measured by DLS (see Methods). The average diffusion coefficient ( $\bar{D}$ ) and the polydispersity index ( PDI ) were obtained from cumulant analysis for the time dependence of the autocorrelation function of scattered light intensities. The first distribution moment ( $\mu_{1}$ ) was estimated from the average intensity weighted diffusivity $\mu_{1}=\bar{D}q^{2}$ , which allowed to obtain the diffusion coefficient $\bar{D}$ from the experimental dependence with the scattering vector q . The second moment ( $\mu_{2}$ ) was proportional to the variance of intensity weighted distribution, i.e., $\mu_{2}=\left(\bar{D^{2}}-{\bar{D}}^{2}\right)q^{4}$ . The polydispersity index was defined as $PDI=1+\mu_{2}/\mu_{1}^{2}$ , being close to unity for nearly monodisperse samples ( PDI≤1.2 ); for broad distributions, we usually find PDI>1.8 . The DLS-measured hydrodynamic size $R_{H}$ , corresponding to an effective particle diameter considering the electric double layer and solvation thicknesses, was estimated by the Stokes–Einstein relationship (Berne and Pecora, 1976); this is $\bar{D}=k_{B}T/6\pi \eta R_{H}$ , where $k_{B}$ is the Boltzmann constant, T is the absolute temperature, and η is the solvent viscosity, assuming sticking conditions between the suspended particle and the solvent. With the synthesis outcome resuspensions (diluted at ca. 0.1 mg/ml), measurements were averaged at least three times with pelleted and supernatant samples, leaving about 30 min between measurements. Table 2 shows the results obtained from the values of three experimental synthesis replicates. For the synthetized LNP samples, the DLS measurements were reproducible and steady along time. For the clodronate formulation, the DLS readout fluctuates largely but steady around a mean value so that measurements resulted in a larger uncertainty. All the samples remained stable along days, as verified by a constant average of scattered light. DLS distributions were found monomodal in all cases, as corresponding to homogenous populations (although differentially broaden at the dependence of polydispersity).

**TABLE 2:** Experimental DLS results for the measurements of the hydrodynamic size and polydispersity index in the diluted phases obtained after colloidal synthesis (GR@LNPs in the pellet and hollow LNPs in the supernatant). For comparison, CLO#GR@liposome data in the last column correspond to liposomal GR formulations based on commercial clodronate (CLO); see Methods.

GR@LNPs in the pellet			Hollow LNP (supernatant)		Clodronate formulation (CLO#GR@liposome)	
Diameter (nm)		Polydispersity index (PDI)	Diameter (nm)	Polydispersity index (PDI)	Diameter (nm)	Polydispersity index (PDI)
1	169 ± 16	1.17	191 ± 24	1.19	340 ± 120	1.54
2	174 ± 18	1.20	194 ± 20	1.24	290 ± 100	1.35
3	173 ± 20	1.17	195 ± 25	1.22	450 ± 200	1.60
Mean	**172 ± 18**	**1.18**	**193 ± 23**	**1.22**	**400 ± 200**	**1.50**

Bold refers to statistically averaged mean values.

The DLS measurements from the pellet fraction (resuspended in PBS buffer) indicated that the GR@LNPs are rather monodispersive, with an average size around 172 ± 18 nm, compatible with the expected emulsion droplet size (Kheirolomoom et al., 2015). The samples measured from supernatant fractions indicated a higher polydispersity and a slightly larger size of about 193 ± 23 nm, which suggests a lighter aggregate condensation than in the GR@LNPs found in the denser pellet. In agreement with the textural TEM characterization (see Figure 5), these DLS results evidence the appearance of compact GR@LNPs in the heavy phase (pellet), whereas the hollow particles separate out in the light phase (supernatant). The CLO#GR@liposomes detected in the foamy clodronate formulation were characterized by a significantly larger size variable in a broad interval 400 ± 200 nm, as corresponding to a high polydispersity due to local variabilities and mesoscopic reorganizations of the foamed structure (also observed by TEM; see Figure 5; bottom). The nanoparticle sizes determined by DLS are apparently larger than those observed by TEM as corresponding to hydrodynamic diameters, including the hydration layer that surrounds the aggregates.

#### 5.2.3 Surface charge density

The zeta potential was also measured for the same diluted suspensions that were used for DLS experiments. We used electrophoretic cuvettes with the addition of 1 mM of KNO_3_ to regulate velocity mobilities (see Methods). In this case, the process was five-fold replicated with pellet and supernatant samples. According to design specifications of the ZetaPlus device, the electrophoretic velocity is the quantitative observable, allowing to estimate the particle mobilities $\mu_{e}$ . The relation between the zeta potential ζ and $\mu_{e}$ depends, however, on the theoretical model. For charged colloidal particles in the viscous drag regime, the Smoluchowski limit is considered $\mu_{e}=\epsilon \zeta /\eta$ ( ϵ is the dielectric permittivity, and η is the viscosity of the suspending solvent; for NPs dispersed in the KNO_3_ solution $\epsilon =100\epsilon_{0}$ and $\eta =10^{-3}Pa.s$ ). Table 3 collects the experimental results obtained for the five replicates of colloidal synthesis outcomes, as mentioned earlier, protocolized to get the considered nanovector constructs.

**TABLE 3:** Experimental values of the ζ potential measured in the colloidal phases obtained after sedimentation of the GR@LNPs in the denser pellet and decantation of the hollow LNPs in the lighter supernatant. Data correspond to five independent experimental series from different synthesis rounds.

ζ potential (mV)			
Synthesis	GR@LNPs in the pellet	Hollow LNPs in the supernatant	Clodronate formulation
1	–2 ± 2	40.7 ± 1.4	–45 ± 12
2	3 ± 2	44.7 ± 1.5	–56 ± 20
3	3 ± 2	42.4 ± 1.6	–26 ± 18
4	–3 ± 2	48.9 ± 1.6	-
5	–4 ± 2	44.3 ± 1.4	-
Average	**0 ± 3 mV**	**44 ± 4 mV**	**−42 ± 20 mV**

Bold refers to statistically averaged mean values.

As a matter of fact, we found a different zeta potential in the pellets obtained from the synthesis rounds than that in the correspondingly recovered supernatants. For the pelleted GR@LNPs, we found the zeta potential with vanishing values, averaging around 0 mV, a mean compatible with the electrical neutrality expected for the stoichiometric GR:DChol_2_ catanionic lipoplexes. In other words, the lipoplex charges seem to be completely counterbalanced upon the Manning-driven assembly, as synthetically forced in the organic BD solvent (maximum chloroform phase). However, the lighter nanoparticles found in the supernatant phase contain less or no GR so that the excess of the cationic lipid (DChol) cause a net charge to be significantly positive, as deduced from the observed ζ potential (44 ± 4 mV in the supernatant). For the clodronate formulation, the electrophoretic measurements were quite variable as occurred in DLS experiments. However, we detected in that case a negative potential as corresponding to anionic CLO#GR@liposomes with a GR payload, endowing them with a negative charge. It is worth recalling that the measured electrophoretic charges correspond to an unscreened “bare” surface potential (as measured in a very diluted medium of low ionic strength; KCl 1 mM ). In biological media, however, a stronger electrostatic screening is expected from a much higher ionic strength ( ≫100 mM ), which leads to an effective decrease in the Coulombic interactions in the cell (Honig and Nicholls, 1995). Consequently, the electrophoretic charges inferred in this work must be considered only with a physicochemical significance in determining the degree of electrostatic neutralization for the studied nanovectors. In the physiological context, however, their meaning is only relative as far as the Coulombic interactions become effectively screened out, hence decreasing by more than one order of magnitude. In particular, the net positive charge measured for the hollow @LNPs due to the excess cationic lipid should be effectively screened out in the physiological cellular milieu.

### 5.3 Morphology and lamellar ultrastructure: Nanovector types

**FIGURE 7:**
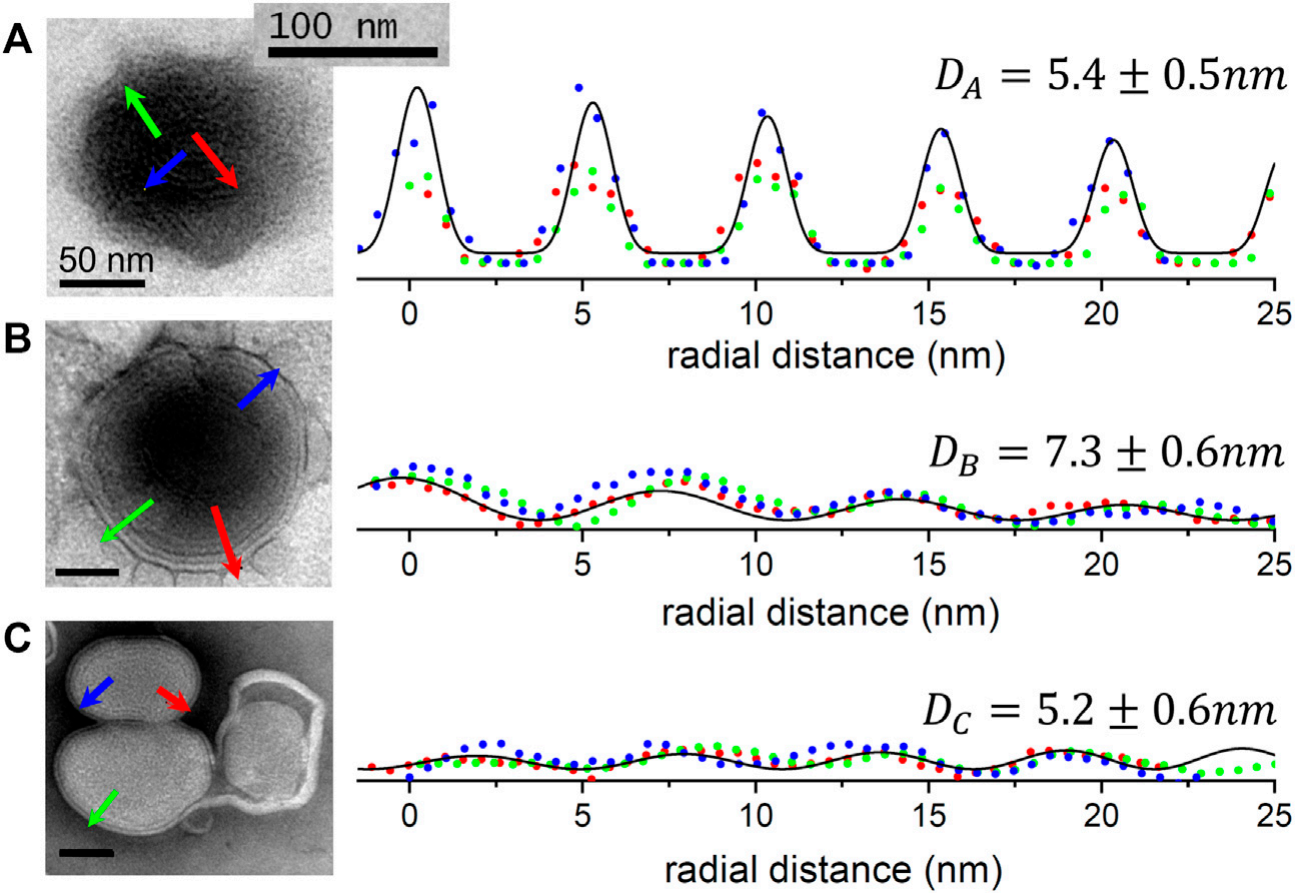
Ultrastructural TEM analysis. *Left panels*: prototypical specimens for the populations of GR@LNPs stratified from the catalog reported in Supplementary Materials (Supplementary Figure S2); the original scale bar is included for reference (100 nm). Colored arrows indicate the vectorized lines chosen for intensity segmentation for quantitative analytic profiling. *Right panels*: profiling analyses for the vectoral segmentations indicated by the arrows (equal colors). The quantitative profiling analysis was performed in terms of the lamellar structure factor and defined the radial distance ( r ); this is $S\left(r\right)=S_{0}e^{-kr}{\sin\left[\pi r/D\left(1+\delta r\right)\right]}^{\alpha }$ (see Supplementary Figure S3 for fitting details, and Supplementary Table S1 for the best fitting parameters). **(A)** GR@LNP compact archetype (*Type A1*). Compact nanoparticle as obtained by GR/DChol complexation with an alternated lamellarity (dense/light/dense/light/…), corresponding to catanionic periodicity (stoichiometric GR:DChol_2_ assembly, as foreseen in Figure 2C). **(B)** GR@LNP core–shell stratified (*Type A2*)*.* Also obtained from the pelleted fraction of the lipoplexing GR/DChol synthesis outcome, the prototypical specimen showed the characteristically stratified ultrastructure expected for a dense dry core constituted by lamellar GR:DChol_2_ lipoplexes (and structurally adjuvant lipids; neutral cholesterol and POPC) and a swollen corona made of functional PEGylated lipids with a more expanded (less compact) bilayer packing (containing the mannosylated ligand and the PEGylated lipid added for improving adhesivity). **(C)** Hollow @LNP (GR-devoid *Type* **(B)** as corresponding to non-complexed nanoparticles recovered from the supernatant fraction of the synthesis outcome (excess unreacted DChol and added adjuvant lipids, especially POPC and neutral cholesterol).

The nanoscopical morphology and the internal structure of the synthetized GR@LNPs were both studied by ultrastructural TEM analysis, inferred from the population catalog supplied as Supplementary Materials (Supplementary Figures S2A, B). We consider two principal types of @LNPs: *Type A*) the GR-containing nanoparticles (GR@LNPs), as obtained from the pelleted fraction of the reaction outcome, were further identified at different degrees of payload compaction (*Type A1:* compact and *Type A2:* core–shell stratified); B) the hollow nanoparticles not containing GR (hollow@LNP), as recuperated from the supernatant fraction from the reaction outcome. Figure 7 shows the main results exemplified with representative specimens for the homotypical populations stratified from the general textural characterization specified earlier (in Figure 5; see Methods). As revealed by uranyl staining (revealing negative charges under electrodensity contrast), the high-resolution TEM images provided the localization of the electron-dense GR polyanions inside the GR@LNPs (Figure 5), which were analyzed in terms of a lamellar structure factor (Supplementary Figure S3). Figure 7 shows quantitative results from the ultrastructural analysis of the lamellar structure factor (performed in terms of radial profiles; right panels). The quantitative results from such TEM characterization were obtained for representative homotypic specimens (as classified in Supplementary Figure S3, with best-fit parameters reported in Supplementary Table T1); by the LNP type, they are classified as follows:

*Type A1*) Compact GR@LNP archetypes, as found in the pelleted part of the synthesis outcome: The lipid nanoparticle shows an electron-dense core with a visible lamellarity (Figure 4A; left panel). Detailed profiling analysis showed a repetitive multilamellar structure (Figure 4A; right panel). The radial fittings to the core–shell structure factor revealed a uniform lamellarity characterized by a repeating spacing $D_{A}=5.4\pm 0.5\;nm$ (associated with the natural radial decay $k_{A}\ll R_{0}^{-1}\approx 0$ and almost zero lamellar dilation $\delta_{A}\approx 0$ ; see Supplementary Figure S2 for details). The repeating lamellar structure is characterized by a high compaction factor ( α≈8 ), which determines the narrow thickness of the electron-dense layer as $d_{A}=0.9\pm 0.4\;nm\ll D_{A}$ . This ultrastructural lamellarity is compatible with the molecular periodicity of catanionic lipoplexes, ideally designed in Figure 2C,for repeating compact layers of GR polyanions ca. 1nm width ( $\approx d\approx D_{GR}$ ), intercalated by cationic DChol bilayers ca. 4 nm thick ( $\approx D_{bil}$ ); this results in the nominal lamellar spacing $D=D_{GR}+D_{bil}=5\;nm$ , in agreement with the experimental value ( $D_{A}\approx 5\;nm$ ), whereas the uranyl-stained GR component of the lamellar structure appeared to be highly electron-dense ( $d\approx D_{GR}\approx 1nm\ll D$ ), and the thicker interlayers corresponding to lipids (and other electron-poor species) appear much lighter (near zero TEM-contrast). This particularly asymmetric lamellarity suggests the heaviest GR compaction together with metallic electrolytes and minimal interstitial water into the thinnest polyanion layers as possible ( $D_{GR}\approx 1nm$ ), which alternate with much thicker lipid bilayers hydrophobically organized at the practical absence of electrolytes and/or interstitial water ( $D_{bil}\approx 4\;nm$ ).

*Type A2*) Core–shell stratified GR@LNPs: The considered GR@LNP specimens appeared sectioned in the equatorial plane at which a swollen corona was distinguishable around the more compact core (Figure 7B; left panel). The radial decreasing contrast indicated lesser peripheric GR than found in the previous *Type A1*. The profiling analysis for *Type A2* GR@LNP’s revealed lamellar periodicity again at $D_{B}=7.3\pm 0.6\;nm$ (Figure 7B; right panel). Such a lighter prototype appears thicker than observed for compact specimens (*Type A1*), being characterized by strong radial decay ( $k_{B}=0.05\pm 0.02\;{nm}^{-1}\gg D_{B}^{-1}$ ) and non-negligible lamellar dilatation along the radial direction ( $\delta_{B}\gg 0$ ); see Supplementary Figure S3 for details. These “swollen” *Type A2* nanovector objects would represent less compact GR@LNP specimens, probably containing more interstitial water and ions, thus allowing the outer layers for expanding as including a lighter corona of PEGylated lipids (both mannosylated and neutral as included to favor, respectively, BAM-specificity and surface adhesion within the GR@LNPs).

These TEM-classified subtypes *A1* and *A2* correspond to the extremal status of lamellar packing within a same class of GR-containing nanovectors. Nevertheless, both subtypes appeared mixed within the pelleted fraction of each synthesis outcome and indistinguishable in terms of size (as determined by DLS) and surface electrostatic charge (as determined by the zeta potential). Hereinafter, we will generically refer to as GR-containing *Type A* lipid nanoparticles, or simply GR@LNPs; these are completely different to the hollow nano-objects found in the supernatant (aka hollow @LNPs).

*Type B*) Hollow lipid nanoparticles devoid of GapmeR: these hollow @LNPs are systematically found in the supernatant fraction of the synthesis outcome. Yet armored with a lamellar ultrastructure, the hollow@LNP specimens of this GR-devoid subtype appeared comparatively less dense than the GR-containing relatives found in the pelleted fraction (see Figure 7C; left panel). They appeared as white objects identifiable with a higher lipid content (see also Supplementary Figure S2). The practical absence of uranyl-stained GapmeR within these lamellar structures induced smooth periodic compacity ( α=1 ), without any sign of core–shell stratification ( $k_{C}\approx \delta_{C}\approx 0$ ). Quantitative profiling revealed lipid periodicity $D_{C}=5.2\pm 0.6\;nm$ (Figure 7C; right panel), which confirms sterol mesogenicity being capable to induce lamellarity even if no GR is retained inside the hollow *Type B* @LNPs ( $D_{C}\approx D_{bil}$ ; see Figure 2C).

Experimental design: synthesis outcomes and nanovectors, as based on the aforementioned protocol for @LNP synthesis (see Figure 4); we performed several reaction rounds using different GR payloads (considered under chemical formulation in Table 1; all of them under lipid mannosylation). Each nanovector construct was synthetized at least in triplicate and subjected to fractioning either as GR@LNPs (*Type A* collected in the pelleted fraction) or as hollow @LNPs (*Type B* recuperated in supernatants). The synthesis outcomes previously stratified in physicochemical terms were batched by reference to the different experimental constructs designed for further *in vivo* evaluation (see Table 4; *Reaction outcomes for evaluation of GR nanovectorization in vivo*)*.* Additionally, the commercial clodronate prepared was considered for reference, as evaluated in previous studies (Kida et al., 1993; Polfliet et al., 2001b; Williams et al., 2001; Schiltz and Sawchenko, 2002; Hawkes and McLaurin, 2009; Steel et al., 2010; Vasilache et al., 2015; Pedragosa et al., 2018; Sayd et al., 2020); the corresponding CLO-containing liposomes were identified as CLO#GR@L (Type C preparation). We designed a rationale for *in vivo* evaluation of the nanovectorization activity, as considering three relevant cell types in the neurovascular unit: PVMs, MGMs, and microglia. The experimental design is summarized in Table 4 with reference to the structural nomenclature established earlier (from Figures 5, 7).

**TABLE 4:** Reaction outcomes for evaluation of GR nanovectorization *in vivo*. The bioactive payloads are differentially considered in different reaction batches under chemical formulation, as in Table 1. The different types of payloads and nanovectors formed as reaction outcomes are considered for differential *in vivo* evaluation. The resulting nanovector type was inferred from the structural characterization (TEM, DLS, and zeta potential). *Type A:* lipid nanoparticles (@LNP); *Type B:* hollow oligolamellar liposomes (@L); and *Type C:* liposome foam.

	Bioactive payload				
*Nanovector construct/vectoring evaluation*	*Antisense GR1*	*Antisense GR2*	*Nonsense GR3*	*No payload*	*Nanovector type*
LNPs/gene silencing	GR1 and GR2@LNP	–	–	*Type A1/A2*	
LNPs/positive control	–	–	GR3@LNP	–	*Type A1/A2*
Liposomes/negative control	–	–	–	Hollow@L	*Type B*
Clodronate/BAM depletion	CLO#GR1 and GR2@L	–	–	*Type C*	

### 5.4 *In vivo e*valuation of GR@LNPs as nanovectors toward BAMs

#### 5.4.1 Specific uptake of GR@LNPs by BAMs presenting the mannose receptor CD206

We first performed triple immunofluorescence assays for detecting the cellular uptake of GR@LNP labeled at the 5′ end with a fluorescein-derived isomer (6-FAM and 6-carboxyfluorescein) being emitted at 488 nm (labeled in green). The mannose receptor CD206 (labeled in red) was considered a specific cellular marker for BAMs, as presented in the surface of the two BAM classes, both in perivascular macrophages (PVMs) and in meningeal macrophages (MGMs). The cell nucleus was counterstained with DAPI (labeled in blue). We used coronal sections of the rat brain prefrontal cortex to find out whether engineered antisense GR1 and GR2@LNPs were capable of specifically targeting toward BAMs presenting CD206 (selectively or not into PVMs or MGMs).

**FIGURE 8:**
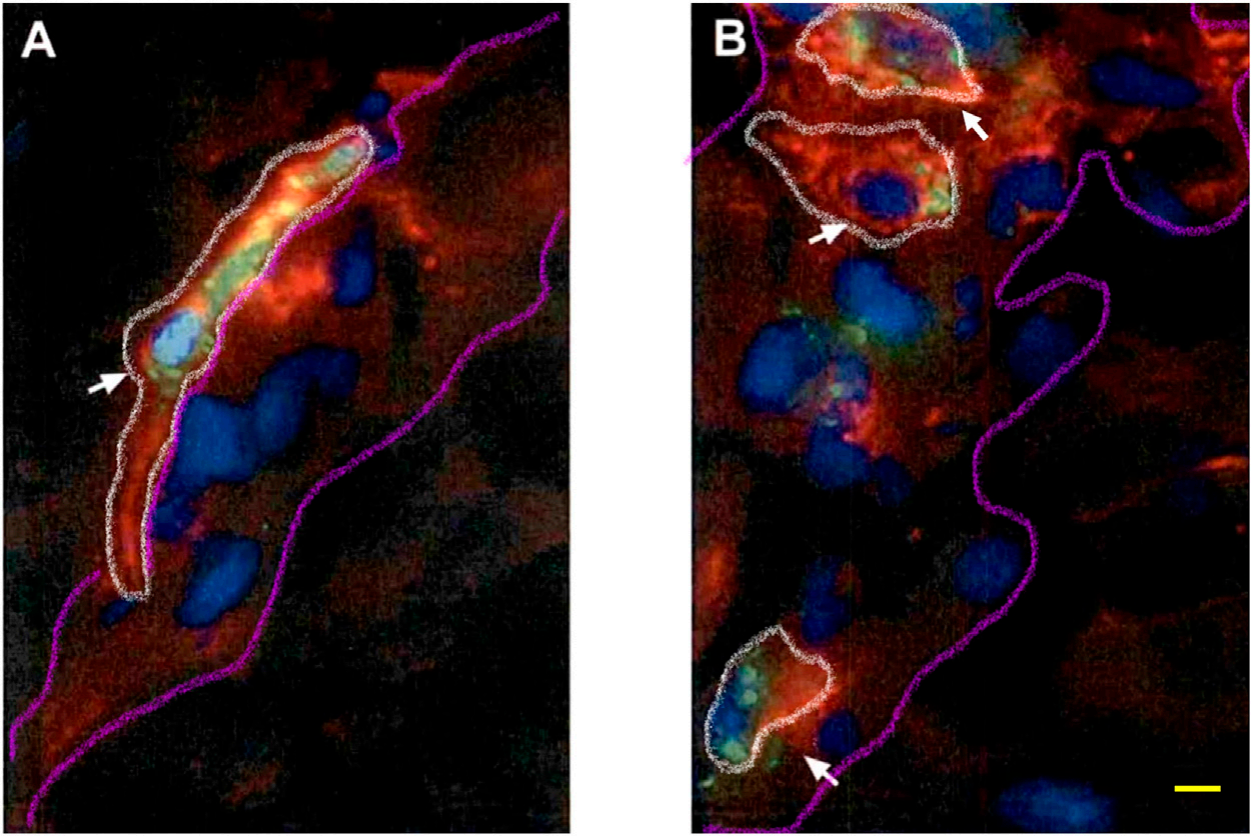
Triple immunofluorescence images of BAMs present in coronal sections of the rat brain tissue. The white arrows indicate the presence of mannosylated GR@LNPs (marked in green) found in perivascular **(A)** and meningeal **(B)** macrophages presenting the mannose receptor CD206 (marked in red). The greenish spots correspond to internalized GR@LNPs standing in the macrophage cytoplasm without association with CD206, whereas the yellowish regions are suggestive of associative colocalization with CD206. The cellular nuclei are marked in blue (DAPI) and have been used to manually and approximately delimitate the outline of the macrophages with shadowed white lines and the blood vessel and meningeal boundaries with curved purple lines, respectively. Yellow scale bar: 5 µm.

Figure 8 shows two representative triple staining-obtained images for perivascular (PVM: Panel A) and meningeal macrophages (MGM: Panel B). The internalized presence of GR@LNPs was revealed in both cases at the perinuclear, cytoplasmic, and membrane levels (albeit apparently more concentrated in PVMs than in MGMs). However, no presence of GR@LNPs was detected in the extracellular spaces of the studied sections, which suggests a complete BAM internalization. The BAM-adopted GR@LNPs appeared mostly in the cytoplasm (as labeled in green), although partially accumulated around the nucleus (labeled in blue). The apparent perinuclear accumulations should correspond to a favored interaction of the catanionic GR@LNPs across the endocytic route. A certain fraction of GR@LNPs appeared yellowish in the triple staining superpositions, as corresponding to colocalized associations within the MAN receptor (labeled in red). Because CD206 is primarily present on the surface of BAMs and immature dendritic cells and not on endothelial cells, these results confirm that the incorporation of GR@LNPs has been specific into the targeted BAMs (white arrows), although it is non-selective for each one of the two locations, either perivascular or meningeal.

#### 5.4.2 Uptake of antisense GR@LNPs into BAM specific marker CD163

**FIGURE 9:**
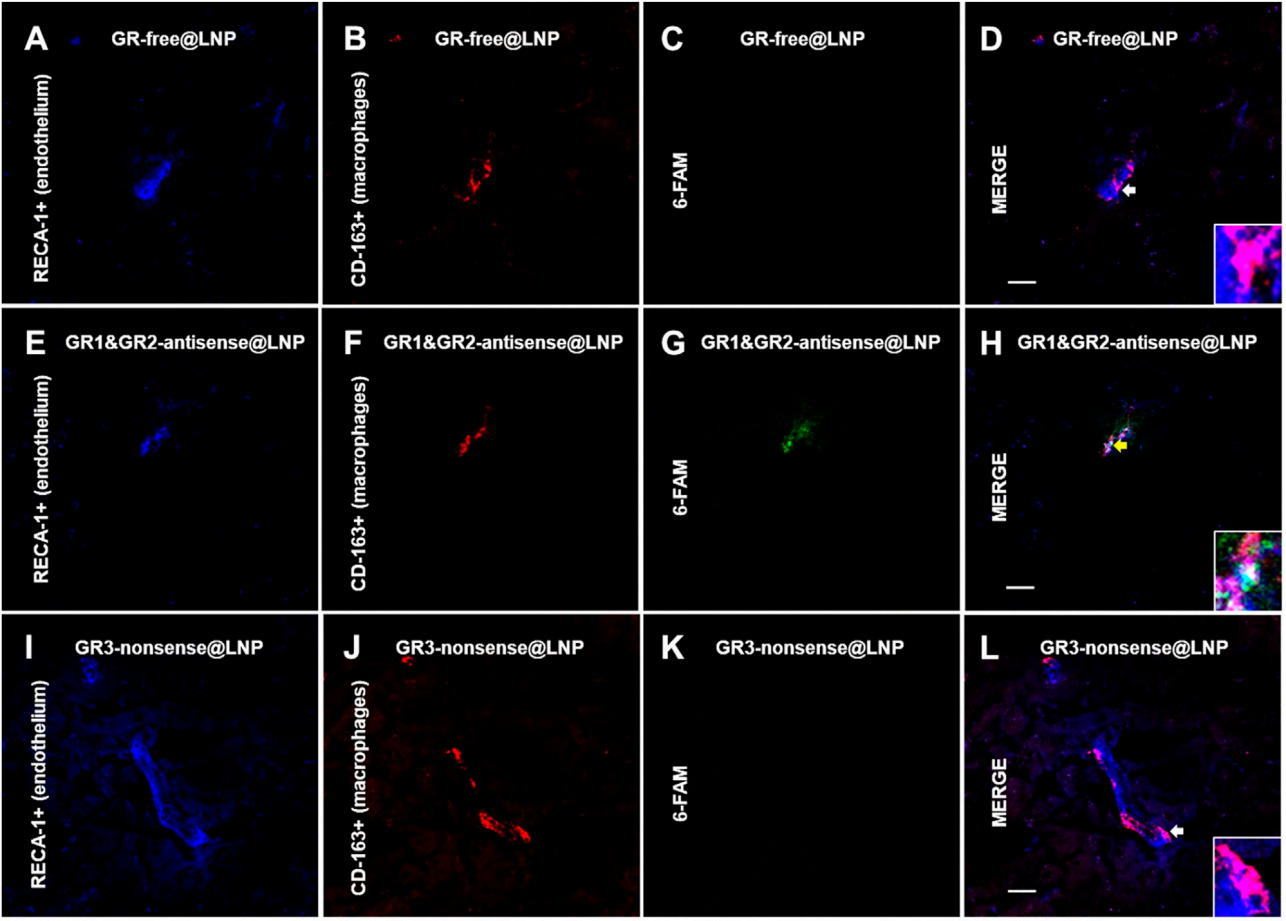
Uptake of GR@LNPs by perivascular macrophages (PVMs). Representative immunofluorescences of 6-FAM-labeled GR@LNPs (green in panels **C**,**G**, and **K**), CD163^+^ perivascular macrophages (red in panels **B**,**F**, and **J**), and RECA-1+ endothelium (blue in panels **A**,**E**, and **I**) in rat prefrontal cortex sections from control rats treated with GR-free@LNP (panels **A–D**), animals treated with GR1 and GR2-antisense @LNPs (panels **E–H**), and negative control rats treated with GR3-nonsense@LNPs (panels **I–L**). Yellow and white head arrows and merge image magnifications, respectively, show apparent (Panel **H**) and absent (Panels **D,L**) co-localizations of GR@LNPs with perivascular macrophages expressing CD-163 in the different experimental groups studied. Scale bars = 15 μm.

**FIGURE 10:**
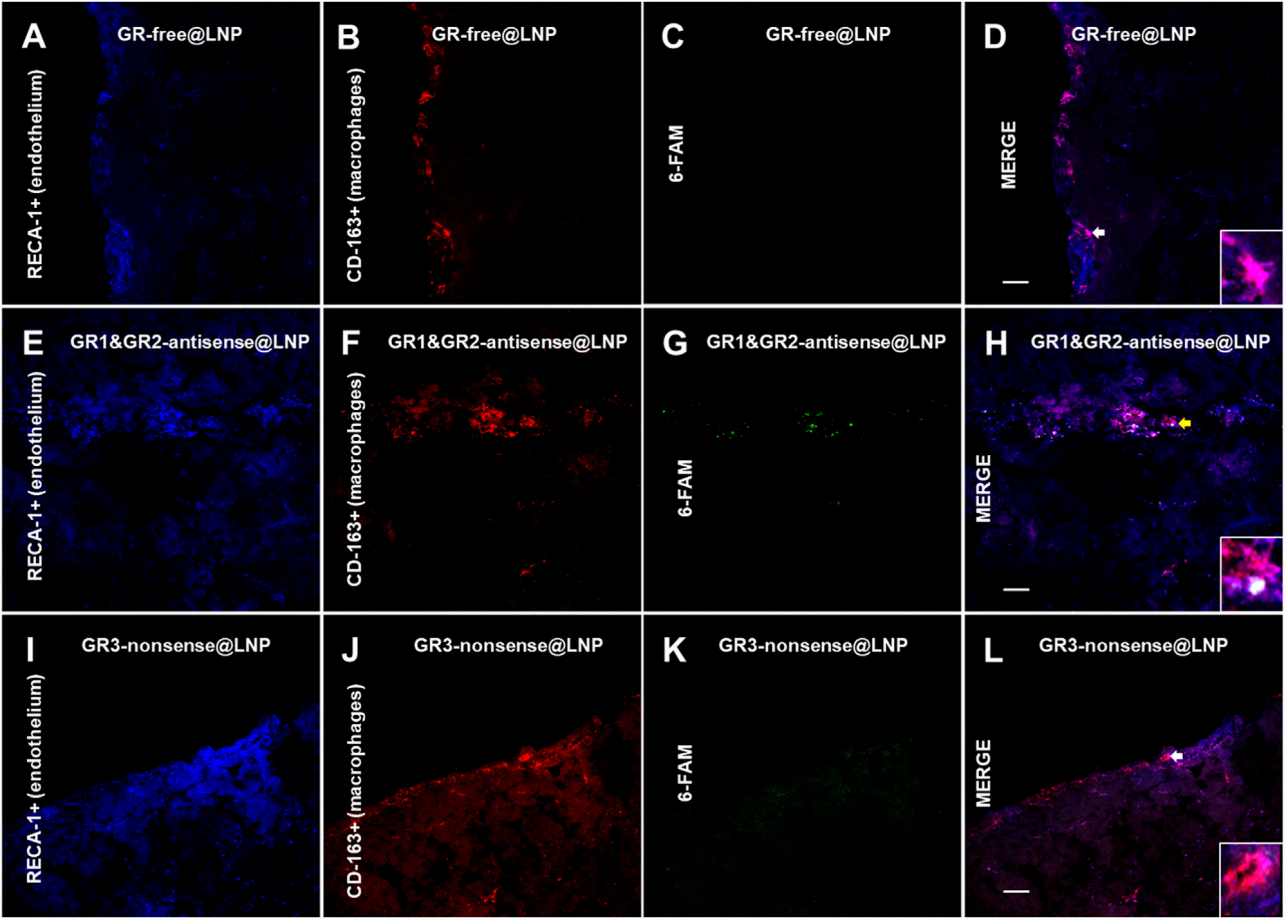
Uptake of GR@LNPs by meningeal macrophages (MGMs). Representative immunofluorescences of 6-FAM-labeled GR@LNPs (green in panels **C,G,** and **K**), CD163^+^ meningeal macrophages (red in panels **B,F,** and **J**), and RECA-1+ endothelium (blue in panels **A,E,** and **I**) in rat prefrontal cortex sections from control rats treated with GR-free@LNP (panels **A–D**), animals treated with GR1 and GR2-antisense @LNPs (panels **E–H**), and negative control rats treated with GR3-nonsense@LNPs (panels **I–L**). Yellow and white head arrows and merge image magnifications, respectively, show apparent (Panel **H**), slightly (Panel **L**), and absent (Panel **D**) co-localizations of GR@LNPs with meningeal macrophages expressing CD-163 in the different experimental groups studied. Scale bars = 15 μm.

To find out whether engineered GR@LNPs were capable for selective targeting into activated BAMs, we further assessed for uptake into PVMs and in MGMs present on coronal sections of the rat brain prefrontal cortex in physiological conditions by confocal microscopy. Immunofluorescence assays were, thus, performed for the presence of antisense GR1 and GR2 labeled at the 5′ end with a fluorescein-derived isomer 6-FAM (6-carboxyfluorescein) being emitted at 517 nm (labeled in green). The high-affinity scavenger receptor for the hemoglobin–haptoglobin complex CD163 was considered the cellular marker for activated BAMs (labeled in red). This specific macrophage protein CD163 identifies the acute phase of inflammation as it endows the property to scavenge hemoglobin by mediating endocytosis under activation during hemolysis (Kristiansen et al., 2001) Additionally, RECA-1 was used to identify the adjacent brain endothelium (labeled in blue). Figures 9, 10 show representative images for perivascular macrophages (PVMs) and for meningeal macrophages (MGMs), respectively. In both figures, the GR-labeled signal (green in Panel G) is present in the group of animals treated with GR1 and GR2-antisense @LNPs (Panels E–H in Figures 9, 10) and localized in a very close proximity to CD163^+^ cells (red in Panel F) and the RECA-1^+^ endothelium (blue in Panels E). As expected, the 6-FAM signal lacks in control rats treated with GR-free@LNP (Type B hollow @LNPs in panels A–D in Figures 9, 10). Unexpectedly, however, it is almost absent in the animals treated with GR3-nonsense@LNPs (panels I–L in Figures 9, 10). This absence of nonsense GR3@LNPs is particularly evident in PVMs (see Figure 4). These results confirm that the GR1/GR2-antisense @LNPs have been predominantly incorporated by the target PVMs (yellow arrows and merge image (H) magnifications) in our experimental conditions. In terms of possible differences *via* electrostatic binding, it is worth remembering that not only the antisense GR1/GR2@LNPs tested but also the nonsense GR3@LNPs that constitute the positive control, and both have in principle the same neutralized surface potential, as corresponding to the catanionic structure.

#### 5.4.3 No GR@LNP uptake occurred in microglia

**FIGURE 11:**
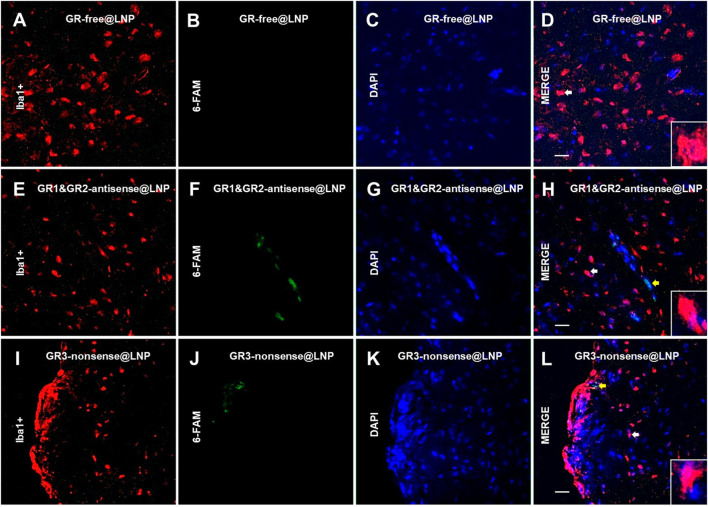
Uptake of GR@LNPs by parenchymal microglia. Representative immunofluorescences of 6-FAM-labeled GR@LNPs (green in panels **B,F,** and **J**), iba1+ parenchymal microglia (red in panels **A,E,** and **I**), and nuclear DAPI (blue in panels **C,G,** and **K**) in rat prefrontal cortex sections from control rats treated with GR-free@LNP (panels **A–D**), animals treated with GR1 and GR2-antisense @LNPs (panels **E–H**), and negative control rats treated with GR3-nonsense@LNPs (panels **I–L**). White head arrows and magnifications in merge images (panels **D,H,** and **L**) show a lack of co-localizations of GR@LNPs with parenchymal microglia in the different experimental groups studied. On the contrary, yellow arrows in H and L show a 6-FAM signal in perivascular and meningeal locations, respectively. Scale bars = 15 μm.

In addition, we also performed immunofluorescence assays to check whether iba1^+^ parenchymal microglial cells were capable to incorporate the different types of GR@LNPs. Figure 11 shows representative images for iba1+ parenchymal microglia (labeled in red) and the respective cellular nuclei (labeled with DAPI in blue). The GR-labeled signal (green in panels B, F, and J) is absent adjacent to parenchymal microglial cells (red in panels A, E, and I) in the three animal groups studied (white arrows and magnifications of merge images (D, H, and L). These experiments confirmed the GR-labeled signal is only present in the group of animals treated with GR1 and GR2-antisense @LNPs (Panel F) and GR3-nonsense@LNPs (Panel J) in perivascular and meningeal locations, respectively (yellow arrows).

#### 5.4.4 Reduction of PGDS mRNA expression

**FIGURE 12:**
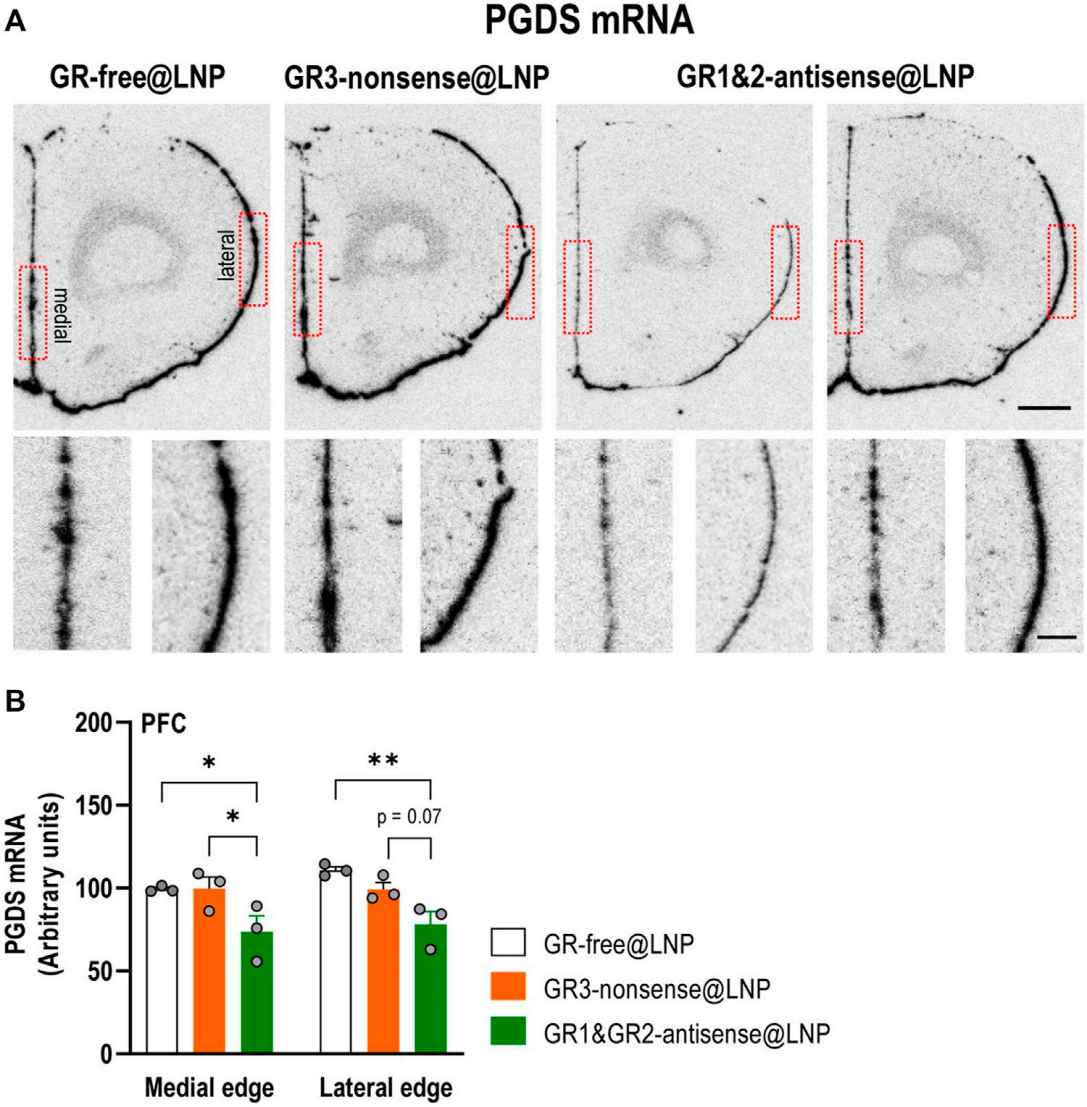
Single administration of GR1 and GR2-antisense@LNP reduces PGDS mRNA expression in peripheral locations of the rat prefrontal cortex (PFC). Rats received intracerebroventricularly (ICV) 25 μL of i GR-free@LNP, ii) GR3-nonsense@LNP, or iii) GR1&GR2-antisense@LNP (total GR concentration estimated at 0.15 nmol/μL) and were euthanized at 1-week post-infusion (n = 3 rats/group). **(A)** Representative coronal brain sections of the prefrontal cortex showing PGDS mRNA levels in brain borderlines. The frames inserted in the images show enlargements from the prefrontal cortex of rats. Scale bars: 1 mm (upper panels) and 0.5 mm (lower panels). **(B)** Significant reductions of PGDS mRNA expression in the medial and lateral edges of the prefrontal cortex of GR1 and GR2-antisense@LNP‐injected rats compared to control rats injected with GR-free@LNP or GR3-nonsense@LNP. The values are presented as mean ± SEM. *p < 0.05 and **p < 0.01, compared to control groups.

Next, to confirm whether administration of antisense GRs reduces PGDS target mRNA expression, an *in situ* hybridization (ISH) procedure was performed with those constructs of Table 4, considering the different functional GR payloads. Rats treated with R1 and GR2-antisense@LNP (ICV 25 μL, total GR concentration estimated at 0.15 nmol/μL) showed reduced PGDS mRNA expression in the medial and lateral edges of the prefrontal cortex compared to control rats treated with GR-free@LNP or GR3-nonsense@LNPs (Figure 12A). Quantitative analysis revealed a significant decrease in R1 and GR2-antisense@LNP-induced PGDS mRNA expression at the medial edges and a marginal effect in the lateral edges of the prefrontal cortex close to 25% (Figure 12B). In statistical terms, two-way ANOVA showed an effect of group *F* (Iadecola, 2017; Sochocka et al., 2017) = 12.84, *p* < 0.001, but not brain locations or interactions group by brain locations (see caption and Supplementary Information for details).

**FIGURE 13:**
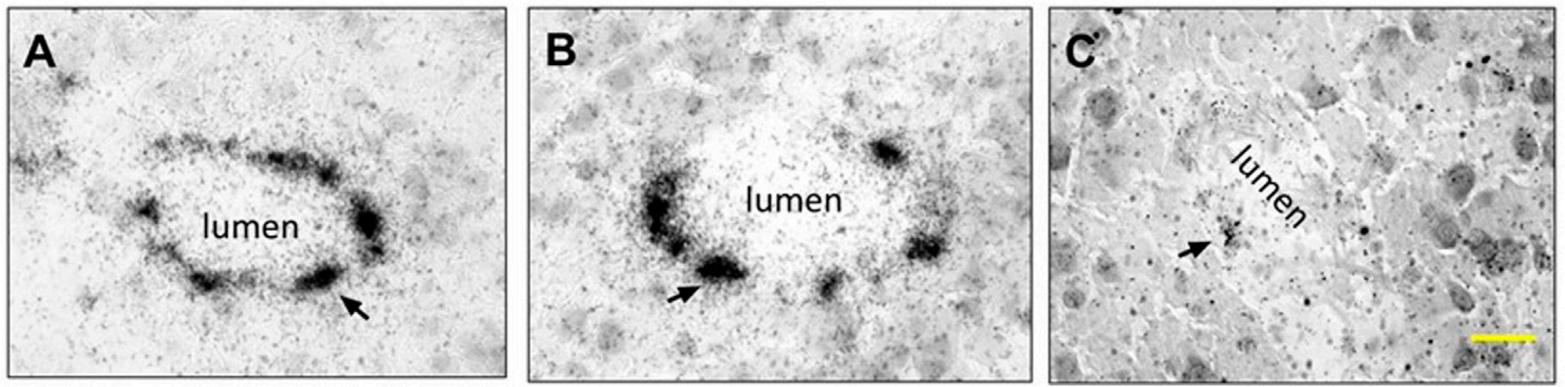
*In situ* hybridization high-magnification images of rat brain vasculature. **(A)** Localization of L-PGDS-mRNA (black grain clusters) in presumable perivascular macrophages (black arrows) around transversal vessel sections (lumen of the brain vessel) of control rats injected with GR free@LNP. **(B)** Negative control group receiving GR@ LNPs with nonsense GR3 (non-complementary to the RNA fragment coding for L-PGDS). **(C)** Positive control group receiving GR@LNPs with antisense GR1 and GR2 (complementary to RNA fragments coding for L-PGDS). Yellow scale bar: 25 μm.

As an additional evidence of GR-driven gene silencing, Figure 13 shows a more refined analysis of PGDS-mRNA expression in high-magnification images of brain vasculature. Furthermore, ISH analysis at higher magnification showed similar PGDS expression in the perivascular brain locations in both control groups (see black arrows in Figure 13; GR-free@LNP in Panel A (hollow@LNP) and GR3-nonsense@LNPs in Panel B). However, PGDS mRNA expression was evidently reduced in the rats treated with GR1 and GR2-antisense @LNPs (Figure 13C). Overall, these ISH data confirm the inhibitory potential of antisense GR-loaded lipid nanoparticles in brain perivascular and meningeal niches.

## 6 Discussion

In the last years, we have been witnessing notable advances in nanomedicine at improving diagnostic imaging and treatments for a wide array of diseases including those affecting the CNS (Silva, 2006; Owen et al., 2014; Ceña and Játiva, 2018; Beltrán-Gracia et al., 2019). Nanotechnological innovations are providing unprecedented assistance in the diagnosis, treatment, and follow-up of patients with brain disorders (Fond et al., 2013; Pampaloni et al., 2018; Nguyen et al., 2021). Different classes of drug cargo nanoparticles (@NPs), including lipid nanoparticles (@LNPs), have been developed for systemic transport of neurotherapy drugs (Pampaloni et al., 2018; Nguyen et al., 2021). Using conventional liposomal approaches to systemic drug delivery, several nanopharmacological products are currently being used as enhancers of conventional medication for CNS pathologies (Sobarzo-Sanchez et al., 2015; Nguyen et al., 2021; Riccardi et al., 2021). Yet, directed nanoengineered CNS delivery is challenging since access to the brain is highly impeded by the BBB wall. Furthermore, once delivered into the brain, the novel nanotherapeutics would further face a challenge that involves targeting specific cell brain populations in the neurovascular units including not only the border-associated macrophages (BAMs, either PVMs or MGMs) but also vascular endothelial cells, neurons, astrocytes, myocytes and pericytes, and associated microglia (Polfliet et al., 2001b; Prinz et al., 2017). The role/s of BAMs in neuropathological conditions have been explored by means of their selective depletion with the ICV administration of the pro-apoptotic clodronate agent included in liposomes (Kida et al., 1993; Polfliet et al., 2001b; Williams et al., 2001; Schiltz and Sawchenko, 2002; Hawkes and McLaurin, 2009; Steel et al., 2010; Vasilache et al., 2015; Pedragosa et al., 2018; Sayd et al., 2020). However, these BAM depleting clodronate-based interventions are also deleterious with other phagocytic cells, including peripheric macrophages and dendritic cells (van Rooijen and van Kesteren-Hendrikx, 2002). Yet, the nanotechnological potential of brain BAMs is enormous not only for pharmacological engineering but also for the fundamental comprehension of the pathophysiology in clinical psychiatry and neurology (Faraco et al., 2017). They could be exploited to intervene the CNS in nanovectorized pathways with a therapeutic significance, including druggable and genetic regulators of neuroinflammation (Prinz et al., 2021).

Although BAMs are not integral components of the vascular walls, they are considered a fundamental part of the neurovascular interfaces due to their interfacial emplacements between vascular lamina and neuroglia limitans (Prinz et al., 2021). Hence, the BAMs are thought to be involved in regulating both, the balance between the proper segregation of the CNS and the essential exchange between the CNS parenchyma and the periphery (Prinz et al., 2021). BAMs constitute an essential part of a complex of brain-infiltrating immune cells, which may be involved in exacerbating or resolving neuroinflammation (Goldmann et al., 2016). Other relevant biological functions have been proposed for BAMs: 1) regulation of hypothalamic–pituitary–adrenal (HPA) axis activation; 2) initiation of fever in response to systemic immune insult; 3) involvement in CNS immune surveillance; and 4) regulation of trafficking of waste macromolecules, e.g., β-amyloid and various microorganisms such as viruses, bacteria, and leukocytes between the periphery and the CNS (Kida et al., 1993; Polfliet et al., 2001b; Williams et al., 2001; Schiltz and Sawchenko, 2002; Hawkes and McLaurin, 2009; Steel et al., 2010; Vasilache et al., 2015; Pedragosa et al., 2018; Sayd et al., 2020). BAMs have also been proposed to be a major source of oxidative stress and cerebrovascular dysfunction even affecting cognition (Faraco et al., 2016; Park et al., 2017; Sayd et al., 2020). Likewise, they produce high levels of the pro-inflammatory cytokine interleukin-1β in the rat subfornical organ in response to circulating bacterial LPS (Morita-Takemura et al., 2019). Particularly, the PVMs may also facilitate lymphatic drainage by several routes (Yang et al., 2019). Hence, nanovectorized gene interferences directed to those biochemical circuitries expressed by BAMs emerge as a new category of the neurotherapeutic strategy.

### 6.1 Mannosylated GR@LNPs directed to BAM targets: A potential CNS nanomedicine

Nanoengineered gene delivery is prominent as exploiting lipid nanoparticles (Frazier, 2015; Novak et al., 2018; Hou et al., 2021). Many BAM-vectorized gene drugs based on ASO/GRs are currently under scrutiny for the maximal gene interference efficiency, minimal off-target binding, and non-toxicity (Frazier, 2015; Huggett and Paisner, 2017; Novak et al., 2018). Despite the variety of gene interferential pathways and expression knockouts available for ASO/GR-based therapeutic intervention nanovectorized into BAMs, a current and growing controversy is on the rise because most of the revealed circuitries were explored under harmful clodronate depletion (Polfliet et al., 2001a; Faraco et al., 2016; Pedragosa et al., 2018; Sayd et al., 2020; Zhang et al., 2021). We have proposed an interferential gene therapy platform based on mannosylated GR@LNPs, as loaded into non-suicidal BAMs. For the sake of validation, we used Wistar rats, in which the targeted BAMs were specifically accessed from the ventricular brain spaces by the ICV route of administration. Immunofluorescence experiments revealed the presence of the mannosylated GR@LNPs in a very specific localization of the perivascular and meningeal macrophages. When delivering antisense GRs into BAMs, their interferential ability to inhibit L-PGDS gene expression was verified by *in situ* hybridization localized in the macrophages of the rat prefrontal cortex. However, no interferential activity was detected with the nonsense GR construction used as a negative control of L-PGDS gene interference. As compared to the conventional clodronate agent formerly used as suicidal GR vehicles toward BAMs (Sayd et al., 2020), an important reduction of L-PGDS mRNA expression was found in the cortical brain areas of rats receiving antisense GR@LNPs.

The mannosylation of the GR@LNPs, as particularly considered in this study with the hydrophobic mannose derivative 4-aminophenyl alpha-D-mannopyranoside, directs the nanovectors to the phagocyte cells of the innate immune system presenting the mannose receptor CD206, including not only the brain BAMs but also other peripheral macrophages (Marichal, 2020; Nielsen et al., 2020). Preclinical checking has evidenced CD206 specificity to improve incorporation of mannosylated liposomes across the BBB (Umezawa and Eto, 1988). Nevertheless, CD206 is also expressed by other non-CNS vascular cells, for instance, liver Kupffer macrophages (Nielsen et al., 2020) or lung interstitial macrophages (Marichal, 2020). In our preclinical setting with model Wistar rats, we opted for testing the mannosylated GR@LNPs, as directed into the rat brain by an ICV injection. Such a rationale assures unambiguous evaluation of interferential gene activity, specifically into BAMs at circumventing off-target binding. Although systemic delivery could have altered L-PGDS expression in multiple rat organs with uncontrolled consequences in the immunity status and indirectly into BAM status, the directed use of GR@LNPs in the brain by the ICV injection follows a more controlled delivery into targeted BAMs. Classical studies have demonstrated indeed that ICV-delivered liposomes containing aminophenyl mannoside were efficiently incorporated into the mouse brain (Umezawa and Eto, 1988). How would mannosylated GR@LNPs be specifically targeted to brain macrophages from systemic delivery is a very challenging question that requests for more extensive and advanced research in the preclinical setting. However, the ICV delivery of the non-suicidal platform in this study developed as GR@LNPs could ultimately offer a chance for better BAM-targeting control than currently achieved with the BAM-depleting clodronate agent. Indeed, the putative specificity of BAM depletion over other cell populations is controversial as currently elicited with mannosylated clodronate-based formulations. In this vein, some authors have stated that resting and inflammatory microglia express CD206 in the rodent brain and are also suitable to be depleted by clodronate-containing liposomes (Marzolo et al., 1999; Zimmer et al., 2003). Other authors conclude that only BAM expresses CD206 in child-like brains and chronic neurodegenerative disease and not microglia (Galea et al., 2005). Our research group and others have found that administration of mannosylated clodronate liposomes did not affect the number of microglial cells in different brain areas of rodents subjected to pathological conditions (Polfliet et al., 2001a; Faraco et al., 2016; Pedragosa et al., 2018; Sayd et al., 2020; Zhang et al., 2021). However, ICV administration of CLO@liposomes with/without mannosylation has been used as an efficient tool to deplete microglia in different experimental conditions, both *in vitro* and *in vivo* (Graykowski and Cudaback, 2021; Yao et al., 2021). Using our mannosylated GR@LNPs (devoid of CLO under the current nanotechnological design), no evidence of GR-immunosignal was, however, found in any parenchymal cellular type other than microglia. Since microglial cells do not need to present an activated profile to phagocyte our GR@LNP carriers, they represent a realistic possibility to further explore neuroinflammatory responses. Therefore, the targeting specificity gained with the mannosylation of GR@LNPs could be further exploited in more advanced/enhanced (pre)clinical scenarios in considering ASO-assisted interventions. Although mannosylation is classically considered to be the most efficient ligand cell-targeting strategy (Umezawa and Eto, 1988), however, the functional role of the CD206 mannose receptor presented by different phagocytic cells might be further assessed by functionally comparing the cellular uptake of mannosylated GR@LNP nanovectors differentially from incorporating the naïve (non-mannosylated) nanoparticles. Different ASO/GR backbones and/or other @GR ligand-conjugations (mannosylated, PEGylated, *etc.*) could also be exploited for BAM-targeted interferential gene delivery.

### 6.2 Nanotechnological GR@LNP proof-of-concept: Colloidal GR compaction into lipid nanoparticles synthesized in Bligh and Dyer solvents

Current advances in gene delivery systems based on lipid nanoparticles have been developed in a variety of chemical synthesis scenarios, including: 1) variable backbone chemistries based on native and/or artificial nucleic acids (Kheirolomoom et al., 2015; Horejs, 2021; Hou et al., 2021; Zhu et al., 2022); 2) tunable self-assembly electrostatics with ionizable lipid moieties (Kheirolomoom et al., 2015; Han et al., 2021); and 3) practicable synthesis solvents with a modulated dielectric permittivity (Duong et al., 2020), including those based on the “green” Bligh and Dyer concept (Breil et al., 2017). Colloidal engineering proposed in this study has provided a synthesis concept for GR@LNP fabrication under modulated dielectric permittivity in conventional BD solvents. Our @LNPs build upon polyanionic ASO(GR) backbones, which become strongly compacted by cationic cholesterol under mesogenic Manning condensation. The catanionic lipid/GR formulation includes additional mannosylated and PEGylated lipids, resulting in a hybrid multifunctional nanovector with an enhancing influence on the physicochemical and biological properties of the pre-structured GRs. A similar B and D-guided synthesis concept was previously exploited to encapsulate prototypical microRNAs for interferential gene delivery toward peripheric vascular endothelia (Kheirolomoom et al., 2015). However, those colloidal RNA nanovectors resulted with a much lighter liposomal vesicle-like structure as mostly composed by an aqueous lumen enclosed by a lipid bilayer coating. A post-insertion methodology was additionally developed to functionalize the outer vesicle leaflet with a PEGylated lipid functionalized with an endothelial cell-targeting peptide. As revealed by TEM, only a few luminal RNA strands were found complexed with the cationic lipid in the inner bilayer leaflet (Kheirolomoom et al., 2015). Such lipid–RNA surface construction is bulky liquid-like as dominated by a water core mostly empty of nucleic acid. This is radically different to our current GR@LNPs with a solid catanionic core supported by a mesogenic skeleton made of nucleic acids complexed into lipid multilayers. The former RNA@liposomal constructs are rather (Kheirolomoom et al., 2015): 1) swollen and comparatively lighter (because of containing more water and less nucleic acid); 2) electrically charged (as no effective Coulombic condensation happens in the aqueous core); and 3) potentially more unstable (because of containing more water). Our GR@LNPs have, however, resulted to be highly compacting, electrically neutral, and internally dry, *better protecting for the nucleic acid payload from water degradation*. As compared to the previous synthesis method in Kheirolomoom et al. (2015), our nano-colloidal aggregates have been claimed with a high loading efficiency and high biochemical stability (patented procedure ES2698565B2).

By exploiting ionizable lipids (other than cationic cholesterol) and greener BD solvents, for instance, newer and optimized GR@LNPs could be designed under colloidal Manning-modulated synthesis. Modulating these physicochemical factors could enhance biocompatibility, tissue penetration, intracellular targeting, and dosing, resulting in fewer toxicities. In nanotechnological engineering terms, a modulable GR-backbone compaction adapted to a programmed pharmacokinetics, and a hidden cationic lipid moiety facilitating lysosomal escape would render into a gene delivery improvement toward the targeted cells. The novel nanomedicines will require compact, stable, safe, and effective @LNPs that protect the nucleic acid from degradation, allowing specific and selective cellular uptake and efficient intracellular release for genetic interference. Our gene delivery approach with ASO/GR into compact @LNP nanovectors can easily be applied to other therapeutics such as bare RNA, DNA, plasmids, and drugs.

### 6.3 Prospections into BAM gene delivery

As a main outcome of our nanotechnological proof-of-concept, a preliminary validation was obtained on the interferential activity of antisense GR payloads into vectorized BAMs as directly accessed from intracerebroventricular spaces. Likewise, other clinically translatable gene therapies were based on interferential ASOs (Novak et al., 2018; Quemener et al., 2020); our GR@LNP nanovectors have been, hence, shown with a pharmacological potential for CNS-targeted ASO delivery. Regarding the route of administration while the ICV injection is occasionally used in the clinics, it is the most often considered suboptimal since it has a high rate of complications and requires extensive medical intervention (Atkinson, 2017; Cohen-Pfeffer et al., 2017). Other more practical routes, such as IV or IP, must be assessed in future translational studies. Our novel nanocolloidal strategy gains biomedical importance, considering that the BAM cellular targets are a bridge between the periphery and brain parenchyma. The proposed nanotechnological approach ought to be chemically bio-orthogonal as it occurs inside the living system without interfering and avoiding chemical injury of brain cells (Silva, 2006; Nguyen et al., 2021). Furthermore, genetic BAM modulation by GR@LNPs can produce changes in other cellular types conforming the neurovascular complexes and probably, in the adjacent parenchymal cells, such as neurons and microglia. Although the present study therapeutically focused BAMs to inhibit the expression of the anti-inflammatory molecule L-PGDS, however, our methodology can be further extended to modulate the expression of any gene that is over activated in other neuroinflammatory/neurodegenerative pathological conditions. At this point, it is worth discussing the unexpected lack of the 6-FAM immunosignal in the positive control group receiving GR@LNPs with nonsense GR3 (non-complementary to the RNA fragment coding for L-PGDS). The reasons for this lack of signal are unknown and could be related to a faster degradation of the nonsense sequence. Indeed, further time-course experiments after ICV administration of our different liposome preparations are needed to shed light into the interpretation of this result. Another important limitation to our immunofluorescence methodology is that it does not allow discriminating BAMs between PVMs and MGMs beyond their different anatomical localization (infiltrating vs. surrounding brain parenchyma, respectively). The discrimination between perivascular and meningeal macrophages is an open and controversial issue in the field.

Recent studies using single-cell sequencing technologies have revealed that MGMs and PVMs are considered as genetically homogenous populations in the homeostatic state, sharing most of their known phenotypic markers and only distinguishable by their specific localization at the CNS interfaces (Kierdorf et al., 2019). Another important limitation to our methodology to be generalized with other ASOs is that we cannot completely rule out the other cellular types in proximity with BAMs in the brain vasculature, such as endothelium, pericytes, or leptomeningeal cells, which are able to incorporate our mannosylated @LNP preparations. This limitation is also extended to our *in situ* hybridization results and could be solved in the future carrying out dual-labeling experiments combining in the same preparations immunolocalization and *in situ* hybridization. Further and complementary studies using high-resolution confocal microscopy would be needed to identify the precise intracellular localization and biological activities of the novel ASO@LNP nanovectors not only inside the targeted BAMs but also in other neurocellular components, both *in vitro* and *in vivo.*

### 6.4 Toward pharmacological optimizing: Formulation and route of administration

Although the new nanotechnological developments provide invigorated interest in ASO/GR interventions, important pharmacological challenges and toxicity issues remain to be addressed with the proposed GR@LNPs to optimize target specificity reducing off-target adverse effects, including 1) the finest route of administration providing better cell-targeting specificity; 2) optimal cell uptake and endosomal trafficking; 3) lysosomal release for enhanced gene interference; 4) minimized proinflammatory effects (vasculitis/inflammatory infiltrates); and 5) negligible (or absent) neurotoxicity under minimal systemic toxicities (mainly nephrotoxicity and hepatotoxicity related to lysosomal accumulation). Further preclinical research requests to elucidate mechanisms for these issues, allowing a better understanding of the clinical relevance of the developed GR@LNPs and pharmacological implications of their toxicities. Pharmacologists, toxicologists, pathologists, and regulatory reviewers need to be familiar with the new ASO/GR@LNP development and their implications. To undergo preclinical toxicity testing, a greater number of ASO/GRs and cationic lipid formulations need to be screened with respect to several cell types targeted from several routes of pharmacological administration. In the therapy context of neuro-inflammation, several authors have tried to selectively deplete BAMs with the intravenous (IV) or intraperitoneal (IP) injections of clodronate-loaded liposomes, and the efficiency was found low, probably because in the systemic circulation, the liposomes have multiple opportunities to be either phagocytosed or extravasated through fenestrated vessels, resulting in a short half-life and rapid degradation in circulation before reaching the CNS (Huitinga et al., 1990; Bauer et al., 1995). The highest BAM-targeting efficiency resulting in a complete depletion of BAM was found indeed with a single ICV injection of clodronate liposomes (Polfliet et al., 2001a; Galea et al., 2005). Nevertheless, the CLO-based liposomes that were not phagocytosed through the ventricular and cisternal spaces of the brain could be retrogradely leaked into the venous blood; consequently, their lost cargoes can eventually affect other cell populations including peripheral macrophages. Therefore, a directed access to CNS phagocytes required the CLO liposomes to be administered directly into the cerebrospinal fluid (Galea et al., 2005; Sayd et al., 2020). Here, we chose the ICV administration as a logical first step to test the success of our nanoformulations to reach BAM targets in the brain, but certainly, the current experimental design cannot discern the differential impact of our GR@LNP nanovectors in different classes of systemic macrophages. However, using ICV administration instead of the IV or IP routes, we have mostly directed the impact of our formulations into BAMs, thus reducing retrograde effects in peripheral macrophages. Therefore, not only the ICV route but also other (systemic and intranasal) routes must be further tested in future preclinical work.

### 6.5 Toward reliable ASO neurotherapies: Strengths and weaknesses

The chemical structure of ASO/GRs is designed to increase the resistance to nuclease degradation and enhance *in vivo* stability. Despite their constitutional stability against endonuclease degradation as extensively determined *in vitro* (Duschmalé et al., 2020), naked ASO/GRs can be degraded *in vivo* into multiple and partially uncontrolled ways (Agrawal et al., 1995; Eder et al., 2009). We thus prevented the naked GRs from non-controllable use in the current proof-of-concept context. Comparatively, our compact GR@LNPs have been objectively shown to be more stable than the liposomal clodronate preparation, which is relatively more stable than conventional liposomes. As compared to naked ASO/GRs, the vesicle liposomes protect the ASO/GR cargoes into the luminal space, hence improving stability in the biological milieu, enhancing cellular uptake, easy escape from the endocytic pathway, and promoting drug distribution (Pakunlu et al., 2006; Zylberberg and Matosevic, 2016). Because the GRs considered in our work are likely to be phagocytosed by multiple cells (endothelial cells, neurons, microglia, and astrocytes), we designed a methodology to specifically reach the macrophages in perivascular and meningeal locations. With respect to previous approaches based on diluted cargoes by luminal carrier liposomes non-specifically targeted under surface-adherent PEGylation (Bunker et al., 2016; Cheng et al., 2022), the novel mannosylated GR@LNP nanovectors compactly charged toward phagocytic brain macrophages are strengthening therapeutic promise for the following reasons: 1) they specifically target the mannose receptor of BAMs; 2) their natural fate is phagocytosis; once internalized, they do not easily escape from the cell; 3) the intracellular release occurs along the endocytic pathway; once inserted in the BAM endocytic way, the lipid components are digested by lysosomal lipases; and 4) they accumulate in perinuclear proximity, favoring GR release into the ribosomes. Consequently, the compact GR-payload into cored @LNPs with a mannosylated corona assures optimal gene interference with respect to conventional liposomes under non-specific cell adherence and lighter loading.

As a main strength from the nanotechnological engineering side, our physicochemical characterization has shown possibilities for synthetic regulation of the GR-compaction status through a modulable formulation within the boundaries established under the conditions for the Manning condensation and the colloidal stability of the resulting catanionic aggregates. Specifically for the mannosylated GR@LNPs validated in this work, we have measured a size around 170 nm (as a hydrodynamic diameter) and an effectively zero electrostatic charge (as compatible with a catanionic maximal condensation). They resulted in an electrically neutral multilamellar aggregate self-assembly as a compact GR-containing nanovector. These very particular synthesis characteristics seem to be the most adequate for the artificial GR@LNPs to specifically target BAMs (without needing to cross the BBB). However, one can be wondering whether other characteristics by design are better (or worse) to this purpose. Specifically, nanoparticle sizes ranging 150–200 nm are expected optimal as far as they are easily endocytable by the targeted BAMs (Manzanares and Ceña, 2020) but refractive to the entry by the tight junctions in the BBB (Ceña and Játiva, 2018). A modulable charge could also play a relevant role in regulating the GR@LNP uptake (Asati et al., 2010; Billiet et al., 2012). In general, the cationic nanovectors are better internalized into the cells due to the cell surface negative charges operating over short distances. However, neutral or negatively charged nanovectors are considered less efficiently internalized (Manzanares and Ceña, 2020). We have shown that a nanoparticle electrostatics effectively neutral seems ideal not only as the consequence of an optimal catanionic condensation in the considered GR@NPLs but also in order to prevent undesired nanovector-BAM repulsions. It is worth mentioning that, independently of the net charges involved, electrostatic screening operates by necessarily unbinding non-specific uptakes because of the high ionic strength stressed by the physiological milieu over long distances. The fact that the neutral GR@LNPs appeared well internalized into BAMs but not into microglia seems quite a matter of specific adhesion interactions mediated by mannosylation and not by non-specific Coulombic interactions.

As a potential biological weakness of the proposed ASO@LNP constructs, mannosylation could become not sufficiently selective or not adequately adherent to phagocytic BAMs but efficiently interfering with other phagocytic cells. The ASOs leaked in the venous circulation could thus eventually interact with other components of the neurovascular unit and ultimately affect other cells by retrograde gene silencing in other unspecific cell populations. However, some research groups have proven that the use of mannosylated liposomes is the most effective way to reach phagocytic cells expressing the mannose receptor (Kong et al., 2012; Belogurov et al., 2013). To discern the differential impact of the GR@LNPs among the other classes of peripherical macrophages, our experimental rationale focuses, thus, intentionally and limitedly to BAMs, as accessed from the ICV spaces. Hence, in effect, to certainly circumscribe conclusions on the efficiency of L-PGDS gene interference on the CNS, we used ICV administration (instead of pharmacologically best-suited IV or -IP routes). Although being pharmacologically suboptimal, the ICV administration of GR@LNPs used in our proof-of-concept has the fundamental potential to modulate gene expression by the venous retrograde impact in several types of peripheral macrophages, expressing the mannose receptor (Kupffer macrophages, lung interstitial macrophages, and circulant monocytes). The degree of the effects will depend on the gene manipulated and the nature of the physiological/pathophysiological process studied (e.g., if BAM infiltration is seen or not seen in the brain parenchyma).

## 7 Conclusion

To the best of our knowledge, the present proof-of-concept validates, preliminarily, a first successful GR@LNP construct for ASO gene interference into BAMs; thus, there has been growing interest for more intense basic research for optimization of the patented procedure and further translational research for prospectively testing in the clinics. Apart from further chemical modifications of the mannosylation status, the physicochemical parameters to be matched to the best BAM selectivity and gene interference efficacy of our antisense GR@LNP methodology consist of a specifically adjusting payload level and compaction degree of the bioactive GR by the cationic lipid, the adequate concentration, size, and net electric charge of the resulting nanoparticles, and the targeting functionality of the nanovector prepared to be delivered together with a bio-engineerable control on the optimal dose and route of administration. More specific cell ligand functionalizations, even alternatives to mannosylation, could be explored with these @LNP formulations to specifically reach other cellular types and subtypes in the brain, e.g., dopaminergic or serotoninergic neurons. Because further developments request on enhanced nanotechnological engineering, the promise of the proposed GR@LNP construction stems on the nano–bio colloidal guiding principles is established here on the Manning condensation concept.

The novel GR@LNP platform performs as advanced ASO compacters, biologically interchangeable for different gene interference agents, and susceptible to easy chemical modification for selective macrophage targeting, particularly those used in the present proof-of-concept to vectorize ASO/GRs into BAMs. The use of the ICV injection in the current validating setting has difficulties to translate in the clinical arena compared to other pharmacologically easier routes of administration, but the scope of the present work did not reach so far yet. In a close future, other formulations, payload compactions, and routes of administration such as intranasal or systemic administration would be tested for pharmacological efficacy of modified ASO/GR@LNPs not only to reach BAMs but also to cross the BBB and eventually to directly reach the brain parenchyma.

As a concluding remark, the proposed ASO@LNPs could emerge across the pharmaceutical technology as promising vehicles to deliver a variety of therapeutics not only as considered in this study for interferential gene control of neuroinflammation but also for other gene therapy approaches to be translated to the personalized management of cancer and infectious diseases. Currently in the spotlight as components of the COVID-19 mRNA vaccines, for instance, @LNPs could play a key role in effectively protecting and transporting mRNA to cells. Advanced cargo @LNPs based on cationic lipid complexes (and particularly with cationic forms of cholesterol), akin a sophisticated version of classical pharmaceutical liposomes, are a versatile nanomedicine delivery platform exhibiting more efficient delivery architecture and pharmaceutically compliant formulations than former versions as neutral liposomes. With their ability to encapsulate and deliver therapeutics, particularly nucleic acid payloads, to specific locations within the body and to release their contents at a desired time, @LNPs provide a valuable platform for the treatment of a variety of diseases.

## Data Availability

The raw data and images presented in this article are included in the published article (and its Supplementary Information files). Requests to access the processed datasets should be directed to the corresponding authors.
