# Supplementary material for: Lipid nanoparticles for antisense oligonucleotide gene interference into brain border-associated macrophages
Source: Front Mol Biosci. 2022 Nov 3;9:887678. doi: 10.3389/fmolb.2022.887678 (PMC9671215; doi:10.3389/fmolb.2022.887678)
Supplement: Supplementary file 1 [file DataSheet1.pdf]

SUPPLEMENTARY MATERIALS

for

Lipid nanoparticles for oligonucleotide delivery into brain border-associated macrophages to silence neuroinflammation-related genes.

M. Calero, L.H. Moleiro, A. Sayd, Y. Dorca, L. Miquel-Rio, V. Paz, J. Robledo-Montaña, E. Enciso, F. Acción, D. Herráez-Aguilar, T. Hellweg, L. Sánchez, A. Bortolozzi, J.C. Leza, B. García-Bueno* and F. Monroy*

*** Corresponding authors:** bgbueno@med.ucm.es; monroy@quim.ucm.es

Table of Contents:

Supplementary Figure S1. Stability control by 1H-NMR spectroscopy check for covalent stability in the synthetized compound DSPE-PEG-MAN as obtained by click-chemistry mannosylation of the succinimide ligand DSPE-PE-NHS.

Supplementary Figure S2A. Catalogue of GapmeR-based lipid nanoparticles (GR@LNPs). Type A) Compact GR-DChol archetype; Type B) Stratified core-shell population

Supplementary Figure S2B. GR@LNPs' catalogue (cont.). Type C) Hollow, non-complexed with GapmeR @LNPs

Supplementary Figure S3. Ultrastructural analysis of the TEM images in terms of the inhomogenous lamellar structure factor.

Supplementary Table T1. Best fit parameters from TEM-ultrastructural analysis performed in terms of the inhomogenous lamellar structure factor.

Source data Fig. 12.

Supplementary Figure S1. Stability control by 1H-NMR spectroscopy check for covalent stability in the synthetized compound DSPE-PEG-MAN as obtained by click-chemistry mannosylation of the succinimide ligand DSPE-PE-NHS (1H-NMR samples dissolved in deuterated chloroform).

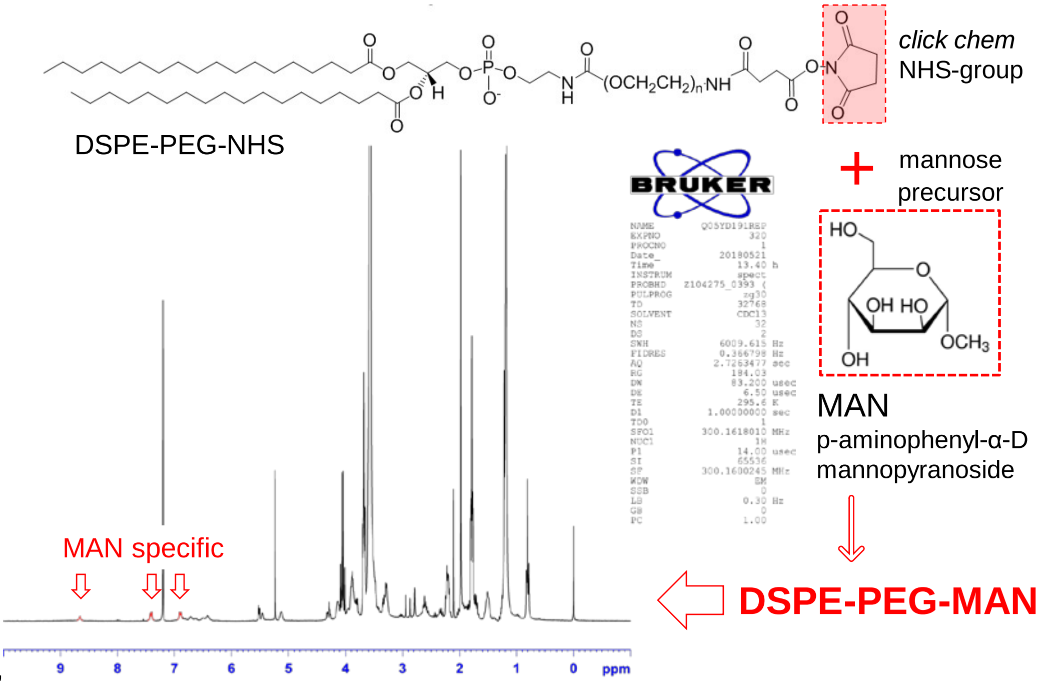

Supplementary Figure S1. Click-chemistry synthesis for the mannosylation reaction used to obtain DSPE-PEG-MAN (covalently reacting moieties framed in red). The proton magnetic resonance spectrum was obtained three months after synthesis of the mannosylated lipid DSPE-PEG-MAN; *inset)* conditions for used RMN sequence. The MAN-specific signals detected after binding p-aminophenyl-α-D mannopyranoside to the pegylated lipid are marked in red. See main text for an interpretation.

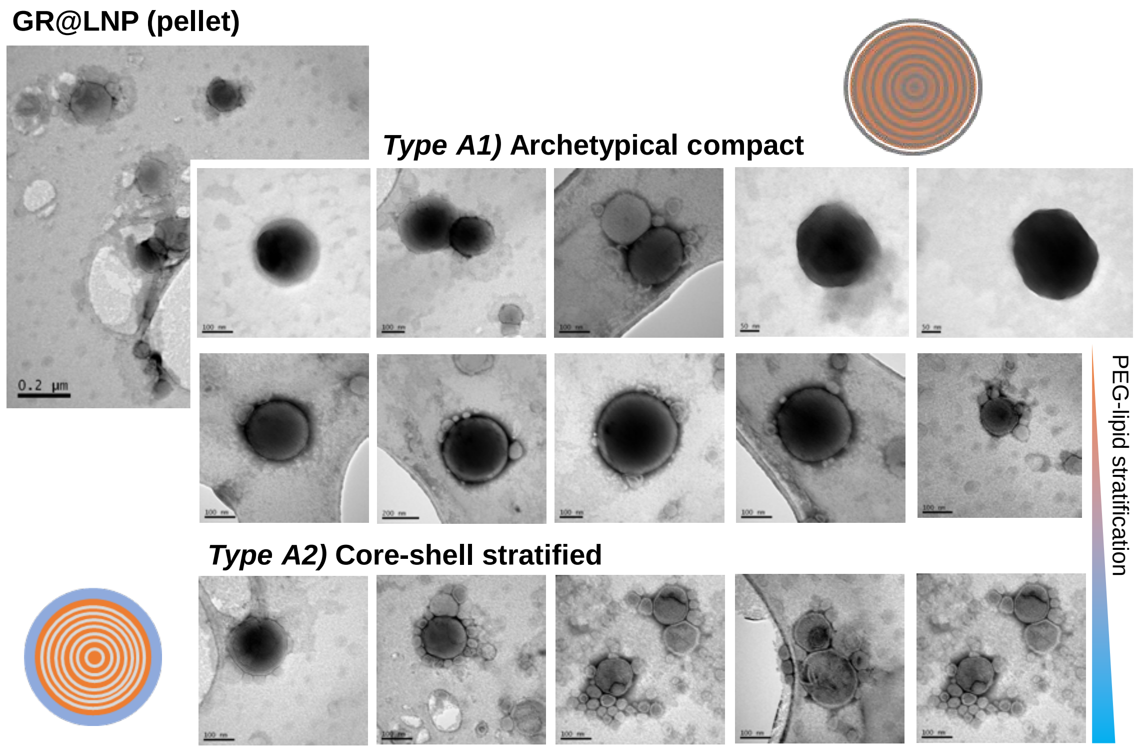

Supplementary Figure S2A. GapmeR based lipid nanoparticles (GR@LNPs) catalogue. *Type A1)* Compact GR-DChol archetype; ***Type A2)*** Stratified core-shell population with swollen corona (in-shell stratified pegylated lipids)

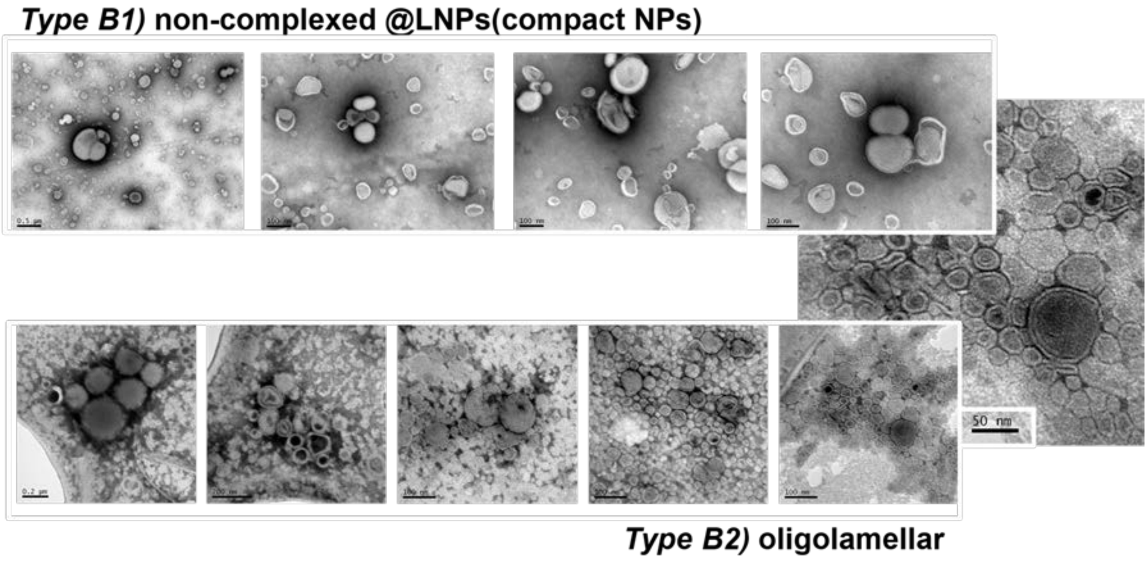

Supplementary Figure S2B. GapmeR based lipid nanoparticles (GR@LNPs) catalogue. *Type B1)* Hollow, non-complexed nanoparticles devoid of the GapmeR component (hollow @LNPs). This typeclass of hollow particles was found in the synthesis supernatants (without evidence of electrodense GR); they were stratified in two subtypes: ***Type B2)*** Multilamellar lipid nanoparticle; *Subtype B2*: oligolamellar, mainly unilamellar liposomes.

Supplementary Figure S3. Ultrastructural profiling analysis of the TEM images in terms of the inhomogenous lamellar structure factor (in terms of the radial distance r; expressed in nanometers):

$$S(r) = S_0 e^{-kr} sin[\pi r/D(1 + \delta r) + \phi]^{\alpha}$$

***<u>Structural lamellarity parameters</u>*:**

- S_0 structural amplitude (in arbitrary units).
- k radial decay factor (in nm^{-1})
- D equivalent wavelength describing the total interlamellar spacing (repetition distance; in nm)
- δ dilatation factor describing core-shell lamellar expansion along the radial direction (dimensionless quantity, relative to the bare wavelength D).
- ϕ phase factor (arbitrary; relative to the initial position of the radial profile).

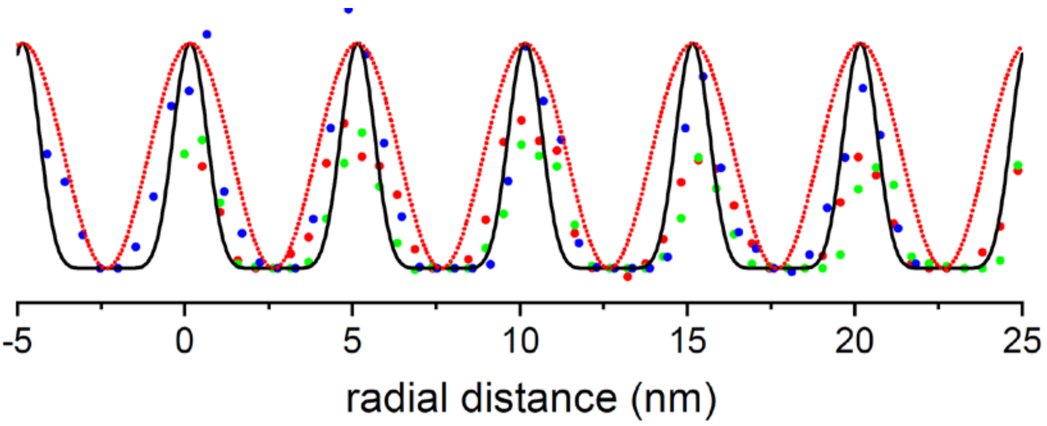

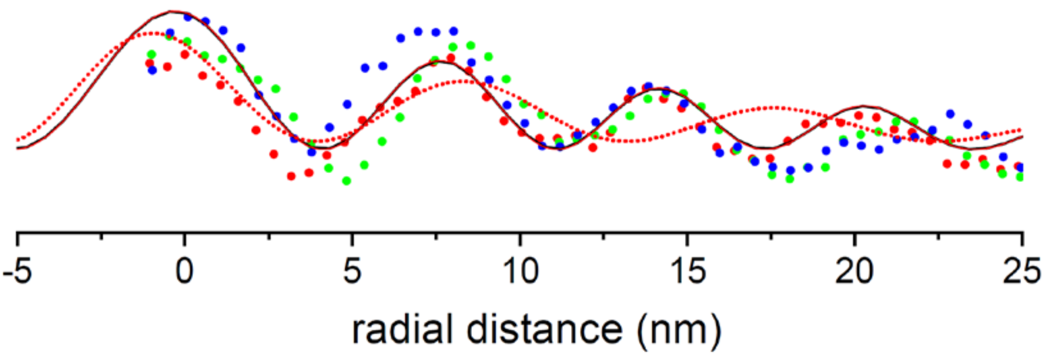

***<u>Legend for Fittings</u>*. Symbols:** experimental intensity profiles as segmented along radial directions in the real nanoparticles (for details, see Fig. 8 in the main text and discussion therein). **Lines:** Best fit to the homogeneous layered model ($\alpha = 2$; dashed line); two-layered alternate model ($\alpha = 8$; straight line).

Supplementary Table T1. Best fit parameters from TEM-ultrastructural analysis performed in terms of the inhomogenous lamellar structure factor.

$$S(r) = S_0 e^{-kr} sin[\pi r/D(1+\delta r)+\phi]^{\alpha}$$

Type	S_0	$k(nm^{-1})$	$D(nm)$	$10^3\delta(nm^{-1})$	$\phi(rad)$	α
A1	2.11 ± 0.16	0.016 ± 0.006	5.02 ± 0.06	20 ± 10	1.47 ± 0.03	8
A2	1.28 ± 0.11	0.020 ± 0.006	5.36 ± 0.08	-14 ± 3	1.54 ± 0.05	8
A3	1.55 ± 0.17	0.027 ± 0.007	5.83 ± 0.08	-28 ± 3	1.95 ± 0.05	8
***A* (average)**	2.47 ± 0.15	0.02 ± 0.01	5.4 ± 0.1	-7 ± 10		8
B1	0.54 ± 0.04	0.05 ± 0.01	7.8 ± 0.4	40 ± 15	1.62 ± 0.09	2
B2	0.71 ± 0.05	0.06 ± 0.01	7.6 ± 0.3	24 ± 10	1.26 ± 0.07	2
B3	0.86 ± 0.05	0.07 ± 0.01	6.7 ± 0.3	14 ± 10	1.31 ± 0.07	2
***B* (average)**	0.78 ± 0.05	0.06 ± 0.01	7.3 ± 0.3	28 ± 12		2
C1	0.17 ± 0.03	-0.02 ± 0.01	6.3 ± 0.4	10 ± 20	0.6 ± 0.2	2
C2	0.17 ± 0.04	-0.004 ± 0.02	4.7 ± 0.4	37 ± 13	2.5 ± 0.3	2
C3	0.41 ± 0.06	0.034 ± 0.01	4.6 ± 0.3	26 ± 15	0.0 ± 0.1	2
***C* (average)**	0.25 ± 0.04	-0.003 ± 0.013	5.2 ± 0.4	24 ± 14		2

Source data Fig. 12.

	Film 1		Film 2		Promedio films 1+ 2		
	**Medial Mean**	**Lateral Mean**	**Medial Mean**	**Lateral Mean**	**Medial Mean**	**Lateral Mean**	
**Rat 1**	100,51	115,17	102,48	113,80	101,495	114,485	CONTROL
**Rat 2**	101,72	102,57	95,03	118,40	98,375	110,485	CONTROL
**Rat 3**	99,05	101,83	97,11	113,15	98,08	107,49	CONTROL
**Rat 4**	89,07	88,37	89,25	80,16	89,16	84,265	GAPMER
**Rat 5**	79,87	85,86	71,60	88,54	75,735	87,2	GAPMER
**Rat 6**	56,52	65,86	54,72	60,11	55,62	62,985	GAPMER
**Rat 7**	95,50	96,82	112,42	90,99	103,96	93,905	NON-SENSE
**Rat 8**	107,63	107,76	109,98	107,63	108,805	107,695	NON-SENSE
**Rat 9**	81,23	92,29	90,86	99,73	86,045	96,01	NON-SENSE
